# Autism throughout genetics: Perusal of the implication of ion channels

**DOI:** 10.1002/brb3.978

**Published:** 2018-06-22

**Authors:** Marwa Daghsni, Mohamad Rima, Ziad Fajloun, Michel Ronjat, Juan L. Brusés, Ridha M'rad, Michel De Waard

**Affiliations:** ^1^ L'institut du Thorax INSERM UMR1087/CNRS UMR6291 Université de Nantes Nantes France; ^2^ Université de Tunis El Manar, Faculté de Médecine de Tunis LR99ES10 Laboratoire de Génétique Humaine 1007, Tunis Tunisie; ^3^ Department of Neuroscience Institute of Biology Paris‐Seine CNRS UMR 8246 INSERM U1130 Sorbonne Universités Paris France; ^4^ Azm Center for Research in Biotechnology and Its Application Lebanese University Tripoli Lebanon; ^5^ LabEx Ion Channels Science and Therapeutics Nice France; ^6^ Department of Natural Sciences Mercy College Dobbs Ferry NY USA; ^7^ Service des Maladies Congénitales et Héréditaires Hôpital Charles Nicolle Tunis Tunisie

**Keywords:** autism, autism spectrum disorder, genetics, ion channels, synapse formation, synaptopathology, therapeutic targets

## Abstract

**Background:**

Autism spectrum disorder (ASD) comprises a group of neurodevelopmental psychiatric disorders characterized by deficits in social interactions, interpersonal communication, repetitive and stereotyped behaviors and may be associated with intellectual disabilities. The description of ASD as a synaptopathology highlights the importance of the synapse and the implication of ion channels in the etiology of these disorders.

**Methods:**

A narrative and critical review of the relevant papers from 1982 to 2017 known by the authors was conducted.

**Results:**

Genome‐wide linkages, association studies, and genetic analyses of patients with ASD have led to the identification of several candidate genes and mutations linked to ASD. Many of the candidate genes encode for proteins involved in neuronal development and regulation of synaptic function including ion channels and actors implicated in synapse formation. The involvement of ion channels in ASD is of great interest as they represent attractive therapeutic targets. In agreement with this view, recent findings have shown that drugs modulating ion channel function are effective for the treatment of certain types of patients with ASD.

**Conclusion:**

This review describes the genetic aspects of ASD with a focus on genes encoding ion channels and highlights the therapeutic implications of ion channels in the treatment of ASD.

## 1 BACKGROUND

For several decades, autism was described as an infantile psychiatric disorder and was termed “childhood schizophrenia.” It was initially believed to be triggered by the psychopathological behavior of the parents and in particular by the behavior of the mother toward her child (Sanua, 1983). In 1943, Leo Kanner characterized for the first time several cases of autism by describing the behavior of children between 2 and 8 years of age (Sanua, 1983). These children were characterized by deficits in social interactions and communication skills including eye contact avoidance, difficulties in understanding the emotions of others, hyper or hypo‐reactivity, focused interests, and stereotyped repetitive behaviors. Because the clinical manifestation of autism may differ significantly among patients, various pathologies were initially described including early infantile autism, childhood autism, Kanner's autism, high functioning autism, atypical autism, pervasive developmental disorder not otherwise specified, childhood disintegrative disorder, and Asperger disorder. This group of disorders is currently encompassed within autism spectrum disorder (ASD; American Psychiatric Association, 2013). The essential clinical manifestations of ASD are persistent in reciprocal social communication and social interaction and restrictive patterns of behavior, interests, or activities. These clinical features may be caused by a variety of genetic abnormalities and environmental factors and may also temporarily overlap with other disorders including Rett syndrome (Park et al., 2016). ASD may be also associated with epilepsy (15%–47% of cases) and intellectual disability (8%–39% of cases; Ko, Kim, Kim, Song, & Cheon, 2016; La Malfa, Lassi, Bertelli, Salvini, & Placidi, 2004).

Genetic causes of ASD were first identified by epidemiological studies of human populations diagnosed with autism (Szatmari, Jones, Zwaigenbaum, & MacLean, 1998). Ozonoff et al. (2011) demonstrated that the prevalence of autism among siblings was 18 times higher than in the general population, suggesting the existence of a familial heritability factor. Also, the imbalance in the sex ratio among ASD cases, with four to five boys affected for each affected girl, has led to the suggestion of a segregation linked to a sex chromosome and the implication of genes whose variations are expressed on a sex‐linked recessive mode (Lai, Lombardo, Auyeung, Chakrabarti, & Baron‐Cohen, 2015; Ozonoff et al., 2011). The second argument in favor of genetic causes of ASD is based on the observation of a change in concordance rate between monozygotic and dizygotic twins, which was found to be 70%–90% in monozygotic twins compared with a lower rate of 0%–30% for dizygotic twins (Ronald & Hoekstra, 2014; Rosenberg et al., 2009). Thirdly, the existence of chromosomal aberrations detected in patients with ASD also points toward genetic causes (Vorstman et al., 2006). Finally, genome‐wide association studies (GWAS) have led to the identification of numerous ASD susceptibility genes that are located on various chromosomes, especially 2q, 5p, 7q, 15q, 17q and on chromosome X (Anney et al., 2010).

In addition, some patients with ASD were found to have variations in syndromic Mendelian genes (e.g., *FMR1* for fragile X syndrome, *TSC1* and *TSC2* for tuberous sclerosis, and *MECP2* for Rett syndrome; Liu & Takumi, 2014). The identification of variations in neuroligin genes (*NLGN4X* and *NLGN3X*) in patients with ASD suggested that proteins involved in synapse formation and synaptic transmission play an important role in the etiology of ASD (Jamain et al., 2003). Similarly, rare variations have been detected in genes coding for ion channels (e.g., *CACNA1* and *CACNB2*), as well as proteins involved in synaptic structure, gene transcription, and chromatin remodeling (e.g., *NRXN1*,* CTTNBP2, CHD8,* and *SHANK3*), indicating that altered synaptic plasticity and regulation of gene expression may also be involved in the etiology of ASD (Cross‐Disorder Group of the Psychiatric Genomics Consortium, 2013; De Rubeis et al., 2014; Durand et al., 2007).

This review examines the genetic basis of ASD and highlights the involvement of ion channel dysfunctions in the causes of this disorder.

## 2 REVIEW

### 2.1 Genetic aspects of ASD

During the last 30 years, a number of reviews have provided detailed description of the genetic architecture associated with ASD (Bourgeron, 2015; Devlin & Scherer, 2012; Li, Zou, & Brown, 2012; Liu & Takumi, 2014; Persico & Napolioni, 2013; Robert et al., 2017). This expansion of knowledge is due to the advances in molecular technologies, which allowed detecting chromosomal rearrangements, copy number variations (CNVs), and candidate genes in patients with ASD.

#### 2.1.1 Chromosomal abnormalities and CNVs in ASD

Chromosomal rearrangements have been identified in 5% of individuals with ASD. These cytogenetic abnormalities are observed in chromosomes 5p15, 15q11–q13, 17p11, and 22q11.2 (Jacquemont et al., 2006; Sebat et al., 2007). Abnormalities affecting the 15q11–q13 region represent the most frequent variation associated with ASD accounting for close to 1% of all ASD cases (Badescu et al., 2016). Depending on the variation type and pattern of inheritance, this locus is associated with either Prader–Willi syndrome (PWS) or Angelman syndrome (AS) along with ASD (Badescu et al., 2016). This variation is based on whether the duplication affects the maternal or the paternal allele (Badescu et al., 2016). Besides structural rearrangements, other abnormalities of chromosomal numbers or aneuploidies are detected in ASD including trisomy 21, Turner syndrome (45, X), and Klinefelter syndrome (47, XXY; Devlin & Scherer, 2012). Thanks to the comparative genomic hybridization (CGH) technique or SNPs array, CNVs were also found in multiple chromosomal regions at 1q21.1, 16p11.2, 17q12, and 22q11.2 (Jacquemont et al., 2006; Marshall et al., 2008; Matsunami et al., 2013; O'Roak et al., 2012; Pinto et al., 2010; Sebat et al., 2007). Further studies supported the association with ASD of two recurrent de novo CNVs at 16p11.2 (duplication and deletion) and 7q11.23 (duplication; Levy et al., 2011; Sanders Stephan et al., 2011). The chromosomal deletion found at 7q11.23 has been linked to William's syndrome, which includes intellectual disabilities, facial dysmorphic features, congenital heart defect, and transient hypercalcemia. The intellectual disabilities suggest that this chromosomal region may also contain genes associated with social behaviors (Pinto et al., 2010). CNVs were found enriched in groups of genes implicated in cell signaling pathways that regulate neuronal development and cell proliferation along with a group of genes associated with the GTPase/Ras signaling pathway and neuronal plasticity (Pinto et al., 2010). CNVs studies demonstrated also that there is alteration in the fragile X mental retardation protein (FMRP) in patients with ASD. In fact, fragile X syndrome (X‐Fra) is associated with 1%–2% of ASD cases. X‐Fra syndrome is the second major cause of intellectual disability, which is caused by the expansion of CGG trinucleotide repeats in the *FMR1* gene located on chromosome X and that encodes FMRP. This protein plays an essential role in synaptic plasticity by regulating mRNA trafficking in the brain (Devlin & Scherer, 2012; Roberts, Tonnsen, McCary, Caravella, & Shinkareva, 2016).

These data emphasized the role of CNVs in ASD, and further investigations in these regions have led to the identification of candidate genes in particular with whole‐exome and whole‐genome sequencing studies.

#### 2.1.2 ASD candidate genes

Next‐generation sequencing (NGS) techniques are very efficient tools for the identification of novel candidate genes associated with ASD. Most of the studies have been performed on sporadic cases of ASD. Using NGS, O'Rack et al. identified de novo variations in *FOXP1*,* GRIN2B*,* SCN1A*,* LAMC3,* and rare inherited *CNTNAP2* variations (De Rubeis et al., 2014; O'Roak et al., 2011). Three genes were found in ASD probands with two de novo variations in each of these genes: *BRCA2*,* FAT1,* and *KCNMA1* (Neale et al., 2012). These studies also found significant enrichment of de novo variations in five ASD candidate genes including *STXBP1*,* MEF2C*,* KIRREL3*,* RELN,* and *TUBA1A* (Neale et al., 2012). Likewise, a region of chromosome 7q that includes the candidate genes *RELN*,* FOXP2*,* WNT2,* and *CADPS2* has been implicated in ASD (Liu & Takumi, 2014). The extracellular glycoprotein RELN plays a key role in neuronal migration and cell interactions (Li et al., 2004). However, it appears that variations in *RELN* are insufficient to cause ASD, suggesting that secondary genetic or epigenetic factors are behind these cases of ASD (Li et al., 2004). *FOXP2* is a crucial gene for language development. Variations affecting this gene have been detected in individuals who lack the ability of acquiring communication skills. However, evidence supporting the involvement of *FOXP2* in ASD remains scattered (Toma et al., 2013). The *WNT2* gene belongs to the large *WNT* gene family, which is highly expressed during development of the central nervous system and, therefore, it is not surprising that it could represent an ASD candidate gene (Kalkman, 2012; Li et al., 2004). Finally, the *CADPS2* gene encodes a calcium (Ca^2+^)‐binding protein and variations in this gene have been linked to patients with ASD and intellectual disability (Bonora et al., 2014). Others genes encoding synaptic proteins linked to ASD were also identified by NGS: They include the glutamate receptors (GRIK2, GRIA3), the cell adhesion molecule CNTNAP2, and the scaffolding protein SHANK3. SHANK3 is involved in (i) synapse formation and maturation, (ii) the link between neurotransmitter receptors and ion channels, and (iii) the interaction with scaffolding proteins and gene regulatory proteins (e.g., protein of chromatin remodeling CHD8; Anney et al., 2010; Cotney et al., 2015; De Rubeis et al., 2014; O'Roak et al., 2011). *NRXN1*,* NLGN3/4X,* and *SHANK3* genes, which encode proteins involved in neuronal cell adhesion and in the regulation of synaptic transmission, are considered strong candidate loci for ASD (Weiss & Arking, 2009). Variations in those loci have also been detected in several patients with ASD (Anney et al., 2010; O'Roak et al., 2011; Table 1).

**Table 1:** Major genes implicated in autism spectrum disorder (ASD)

Gene	Name	Cytogenetic location	Protein function	Associated pathologies	References
*CHD8*	Chromodomain helicase DNA‐binding protein 8/autism susceptibility 18 (AUT18)	14q11.2	Transcriptional repressor negatively regulates Wnt signaling pathway by binding to beta‐catenin thereby inhibiting binding to TCF4	ASD	Cotney et al. (2015), Krumm, O'Roak, Shendure, and Eichler (2014), and O'Roak et al. (2012)
*CNTNAP2*	Contactin‐associated protein‐like 2/autism susceptibility 15 (AUT15)	7q35–q36	Protein member of the neurexin superfamily involved in neural and glia interactions and clustering of potassium channels in neurons	Epilepsy, Pitt–Hopkins‐like syndrome 1, ASD	O'Roak et al. (2011) and Toma et al. (2013)
*CTTNBP2*	Cortactin‐binding protein 2	7q31.31	Modulates the mobility of cortactin in neurons. Regulates spine morphogenesis and synaptic signaling via PP2A complex	ASD	Cross‐Disorder Group of the Psychiatric Genomics Consortium (2013)
*FMR1*	Fragile X mental retardation protein	Xq27.3	FMRP is an RNA‐binding protein involved in RNA translation in neurons	Fragile X syndrome, ASD	Devlin and Scherer (2012) and Roberts et al. (2016)
*MECP2*	Methyl‐CpG‐binding protein 2	Xq28	Chromatin‐associated protein that regulates gene transcription. It is required for the maturation of neurons	Rett syndrome, mental retardation X‐linked syndromic 13, autism susceptibility X‐linked 3	Devlin and Scherer (2012) and Liu and Takumi (2014)
*NLGN3*	Neuroligin 3	Xq13.1	Linked only to glutamatergic postsynaptic proteins	Asperger syndrome susceptibility, autism susceptibility X‐linked 1	Jamain et al. (2003)
*NLGN4*	Neuroligin 4	Xp22.32–p22.31	Binds to neurexins and localized in dendritic spines	Mental retardation X‐linked, Asperger syndrome susceptibility X‐linked, autism susceptibility X‐linked 2	Jamain et al. (2003)
*NRXN1*	Neurexin 1	2p16.3	Neurexins, including NRXN1, are cell surface receptors that bind neuroligins to form a Ca^2+^‐dependent neurexin/neuroligin complex at synapses in the central nervous system. This complex is required for neurotransmission and is involved in the formation of synaptic connexion	Pitt–Hopkins‐like syndrome 2, schizophrenia, ASD	Anney et al. (2010) and Girirajan et al. (2013)
*PTEN*	Phosphatase and tension homolog	10q23.31	Tumor suppressor involved in PI3K signaling pathway and negatively regulates the MAPK pathway	PTEN hamartoma tumor syndrome, macrocephaly, autism	McBride et al. (2010) and O'Roak et al. (2012)
*SHANK3*	SH3 and multiple ankyrin repeat domains 3	22q13.33	Scaffold protein abundant in postsynaptic excitatory synapses where it organizes receptor signaling (e.g., NMDA receptor, mGluR)	ASD, Phelan–McDermid syndrome, schizophrenia	Durand et al. (2007) and Yi et al. (2016)
*SYNGAP1*	Synaptic Ras GTPase‐activating protein 1	6p21.32	Ras GTPase‐activating protein that is largely localized in dendritic spines in neocortical pyramidal neurons. Suppresses signaling pathways linked to NMDA receptor (NMDAR)‐mediated synaptic plasticity and AMPA receptor (AMPAR)	Mental retardation, ASD	Pinto et al. (2010)
*TSC1*	Hamartin	9q34.13	Interacts with tuberin to form a complex that inhibits signal transduction to the downstream effectors of the mammalian target rapamycin pathway (mTOR). Implicated in cell proliferation inhibition	Tuberous sclerosis‐1	Devlin and Scherer (2012) and Liu and Takumi (2014)
*TSC2*	Tuberin	16p13.3	Acts as a chaperone for hamartin protein	Tuberous sclerosis‐2	Devlin and Scherer (2012) and Liu and Takumi (2014)

These approaches have also identified variants in genes encoding ion channels. Here, we describe these variations and highlight the role of ion channels in ASD.

### 2.2 Ion channels and ASD

#### 2.2.1 Calcium signaling and voltage‐gated Ca^2+^ channels in ASD

Ca^2+^ channels are present in many different cell types and they mediate Ca^2+^ influx in response to stimuli which can be a response to (i) change in the membrane depolarization; known as voltage‐gated channels or (ii) to a ligand‐mediated activation (e.g., ryanodine receptor (RyR), inositol triphosphate receptor (IP3R) in the reticulum). In the brain, the elevation of intracellular Ca^2+^ concentration activates several signaling pathways that regulate important neuronal functions such as synaptogenesis, neuronal differentiation, and cell migration (Krey & Dolmetsch, 2007). Dysfunctions of these pathways are responsible for abnormalities observed in patients with ASD, which include an increased cell density, changes in neuronal size, dendritic and axonal branching alterations, as well as in neuronal connectivity (Krey & Dolmetsch, 2007). Voltage‐gated Ca^2+^ channels are devised in two categories: high‐voltage‐activated channels (HVA) and low‐voltage‐activated channels (LVA). HVA include L‐type, the neuronal N‐, P/Q‐, and R‐type. The low‐voltage‐activated Ca^2+^ currents are represented by T‐type channels. HVA are composed by a principal transmembrane subunit α1 (Cav α) associated with a disulfide‐linked α2δ (Cav α2δ) dimer, an intracellular β subunit (Cav β), and a transmembrane γ subunit (Cav γ), while LVA channels are composed only by α1 subunit. Both of HVA and LVA channels control the passive flow of Ca^2+^ across membranes. Therefore, alteration in their components leads to defective channel function that translates themselves into a variety of neurological disorders including hemiplegic migraine, episodic and spinocerebellar ataxia, epilepsy, and ASD (Bidaud, Mezghrani, Swayne, Monteil, & Lory, 2006; Breitenkamp, Matthes, & Herzig, 2015; Heyes et al., 2015; Parellada et al., 2014; Stary et al., 2008; Zamponi, 2016).

The Timothy syndrome (TS) is a channelopathy described to be associated with ASD. TS is a multisystem disorder characterized by autistic features, cardiac abnormalities (QT prolongation), defective immune response, and syndactyly (Splawski et al., 2004). Variations affecting the gene encoding the pore‐forming α_1_ subunit of L‐type voltage‐gated Ca^2+^ channels are associated with TS. Two genetic variations (G406R and G402S), affecting exon 8 of the Ca_v_1.2 channel α_1_ subunit gene (*CACNA1C*), have been associated with TS. They impair the Ca_v_1.2 inactivation and lead to prolonged channel opening and consequent increase in Ca^2+^ flux (Barrett & Tsien, 2008; Splawski et al., 2004). Whole‐exome sequencing of a male patient affected with TS revealed a novel variation in *CACNA1C* gene (p.I1166T; Boczek et al., 2015). Electrophysiological analysis of HEK‐293 cells expressing this gene variant showed a shift in the peak channel activation and a reduced current density (Boczek et al., 2015; Table 2). L‐type channels are predominantly expressed in the heart and brain. They are localized at dendrites and cell bodies of mature neurons and regulate neuronal excitability and Ca^2+^‐dependent signaling cascades involving cAMP‐binding protein (CREB) and myocyte enhancer factor 2 (MEF2; Krey & Dolmetsch, 2007; Simms & Zamponi, 2014). *CACNA1C* plays a key role in the development and functionality of the central nervous system by modulating gamma‐aminobutyric acid (GABA) transmission and influencing neuronal firing. In fact, mice with dysfunctional *CACNA1C* show defects in *N*‐methyl‐d‐aspartate (NMDA) receptor activity leading to an NMDA‐independent long‐term potentiation in the CA1 region of the hippocampus that produces an acute decline in memory. These observations indicate that *CACNA1C* may play a role in NMDA receptor‐dependent signaling and in synaptic plasticity in the hippocampus. In addition to ASD, SNPs in *CACNA1C* gene are linked to psychiatric disorders including schizophrenia and bipolar disorder (Li et al., 2015; Moosmang et al., 2005). In a large GWAS, two genes encoding the α_1_ subunit of calcium channel (*CACNA1C)* and its regulatory β_2_ subunit (*CACNB2)* were strongly linked to psychiatric disorders and ASD (Cross‐Disorder Group of the Psychiatric Genomics Consortium, 2013).

**Table 2:** Impact of genetic variations associated with autism spectrum disorder (ASD) on ion channel's function

Ion channels	Genes	Variations	Type of variation	Impact on ion channel	References
Ca^2+^ channels	*CACNA1C*	p.I1166T	Missense	Shifts peak channel activation and reduces current density	Boczek et al. (2015)
	*CACNA1D*	p.A749G; p.G407R	Missense	Changes kinetics of activation and inactivation	Pinggera et al. (2015)
	*CACNA1F*	p.I745T	Missense	Shifts channel inactivation ~30 mV and significantly slows the inactivation kinetics	Hemara‐Wahanui et al. (2005)
	*CACNA1H*	p.R212C; p.R902W, p.R1871Q/p.A1874V; p.W962C	Missense	All these mutations reduce current density and voltage‐dependent gating properties	Splawski et al. (2006)
	*CACNB2*	p.G167S; p.S197F;p.F240L	Missense	G167S and S197F increase the sensitivity of voltage‐dependent inactivation, and F240L shows an accelerated time‐dependent inactivation	Breitenkamp et al. (2014)
K^+^ channels	*KCNMA1*	9q23/10q22	Translocation	Reduces the activity of the BK_Ca_ channel	Laumonnier et al. (2006)
	*KCNB1*	p.I199F	Missense	Induces partial loss of function relative to biophysical defects of assembled homotetrameric and heterotetrameric channels	Calhoun et al. (2017)
	*KCNQ3*	p.P574S	Missense	Reduces potassium current amplitude	Gilling et al. (2013)
Na^+^ channels	*SCN2A*	c.476+1G>A	Splicing	Produces a nonsense mRNA and a truncated protein which alters the channel properties	Tavassoli et al. (2014)

In addition, three rare missense variations of *CACNB2* (G167S, S197F, and F240L) were identified in families with ASD (Breitenkamp et al., 2014). Heterologous expression of these gene variants in HEK‐293 cells followed by electrophysiological analysis showed remarkable changes in channel kinetics characterized by an increased sensitivity of voltage‐dependent inactivation for both G167S and S197F variants. Unlike these variations, the third variation F240L showed a significant accelerated time‐dependent inactivation (Breitenkamp et al., 2014; Table 2). A deletion in chromosome region 12p13.33, that affects both the *CACNA1C* and the *CACNA2D4* genes coding for the α_1_ channel‐forming subunit and the α_2_δ_4_ auxiliary subunit, respectively, was observed in patients with autistic manifestations (Smith et al., 2012). A chromosomal translocation of 2p:12p resulting in a deletion of both genes (*CACNA1C* and *CACNA2D4*) was detected in two ASD‐affected individuals (Smith et al., 2012). Furthermore, a whole‐exome sequencing study identified de novo rare alleles in α_1_ subunit loci *CACNA1D* and *CACNA1E* (O'Roak et al., 2012; Pinggera et al., 2015). The α_1_ subunit (Ca_v_1.3) of L‐type channels plays an important role in neuronal signaling and in brain function including memory and behavior (Pinggera et al., 2015). De novo variations in Ca_v_1.3 subunit (*CACNA1D*) were identified in a cohort of patients affected with autism along with intellectual disability (Pinggera et al., 2015). Using heterologous expression of the mutant proteins in tsA‐201 cells and whole‐cell patch‐clamp electrophysiological recordings revealed that these genetic variations affect the gating properties of the channel by changing the voltage‐dependent kinetics of activation and inactivation (Pinggera et al., 2015; Table 2). A relevant study based on the analysis of signaling pathways implicated in ASD etiology of 1000 individuals with ASD from the Autism Genetic Resource Exchange (AGRE) identified SNPs in 146 genes. From 15 high‐risk SNPs linked to ASD found in the study, two of them are found in *CACNA1A* encoding Ca_V_2.1 of Ca^2+^ channel and in *CACNA2D3* gene encoding for α_2_δ_3_ subunit of voltage‐gated Ca^2+^ channel (Skafidas et al., 2014). A recent study in Chinese Han population reported for the first time the association of two markers (rs7249246 and rs12609735) in *CACNA1A* gene with patients with ASD (Li et al., 2015). A variation in *CACNA1F* gene encoding for Ca_V_1.4 of Ca^2+^ channel was detected in a family with inherited night blindness and ASD. This variation leads to the substitution of threonine by an isoleucine residue at codon 745 (p.I745T). Functional analysis of this variation performed in tsA‐201 cells demonstrated that it affects channel kinetics causing the inactivation of the Ca^2+^ current (Hemara‐Wahanui et al., 2005; Table 2).

T‐type voltage‐gated Ca^2+^ channels are known to play a key role in the cerebral cortex and in the thalamus (Simms & Zamponi, 2014). Four heterozygous missense variations in *CACNA1H* gene, encoding the Ca_v_3.2 subunit of T‐type channels, were found associated with decreased channel activity in six of 461 autistic patients. This decrease could be a result of abnormal trafficking of the channel (Splawski et al., 2006; Table 2). Variations in the *CACNA1H* gene have also been associated with childhood absence epilepsy (Splawski et al., 2006). Another α_1_ subunit of the T‐type calcium channel‐encoding gene (*CACNA1G*) mapped at 17q11–q21 region was found to contain SNPs (rs12603122, rs757415, rs12603112, and rs198547) in patients with ASD (Lu, Dai, Martinez‐Agosto, & Cantor, 2012). A statistical re‐analysis of a GWAS data of patients with ASD from the AGRE revealed the association of the *CACNA1I* gene encoding for Ca_V_3.3 of Ca^2+^ channel in ASD (Hussman et al., 2011). Yatsenko et al. studied 20 unrelated children affected with neurodevelopmental impairments, speech delay, and ASD using array‐comparative genome hybridization and detected a duplication in 9q34. This region contains the *CACNA1B* gene, and the 3′ region of *EHMT1* gene implicated in Kleefstra syndrome, which is a genetic disorder characterized by intellectual disabilities, infantile hypotonia, severe delay in expressive language, and facial dysmorphism associated with other clinical signs. However, this duplication was described by the author as “benign” because it was also found in the control individual (Yatsenko et al., 2012; Table 3).

**Table 3:** Ion channel genes implicated in autism spectrum disorder (ASD) and related pathologies

Genes	Name	Cytogenetic location	Description	Associated phenotypes	References
*CACNA1A*	Alpha‐1A subunit of P/Q‐type Ca^2+^ channel	19p13.13	Modulates the biophysical properties of P/Q‐type Ca^2+^ channel in neurons	Autism, Asperger or PDD‐NOS, Ataxia, Migraine	Breitenkamp et al. (2015) and Skafidas et al. (2014)
*CACNA1B*	Alpha‐1B subunit of N‐type Ca^2+^ channel	9q34.3	Modulates the biophysical properties of N‐type Ca^2+^ channelwhich controls neurotransmitter release from neurons	Neurodevelopmental impairments, ASD, speech delay	Breitenkamp et al. (2015) and Yatsenko et al. (2012)
*CACNA1C*	Alpha‐1C subunit of L‐type Ca^2+^ channel	12p13.33	Plays an important role in the development of the central nervous system and it functions, especially NMDA receptor function in the hippocampus. The mutation is also implicated in defective synaptic plasticity	Timothy syndrome, psychiatric diseases (bipolar disorder, schizophrenia), Brugada syndrome, ASD	Cross‐Disorder Group of the Psychiatric Genomics Consortium (2013) and Li et al. (2015)
*CACNA1D*	Alpha‐1D subunit of voltage‐gated Ca^2+^ channel	3p21.1	Contributes to different brain functions, such as emotions, memory, and drug dependence. Controls gating and current properties and is involved in pacemaker current	Sinoatrial node dysfunction and deafness, psychiatric diseases, ASD	Pinggera et al. (2015)
*CACNA1E*	Alpha‐1E subunit of R‐type Ca^2+^ channel	1q25.3	Modulates the biophysical properties of R‐type Ca^2+^ channel	ASD, psychiatric diseases	Lu et al. (2012)
*CACNA1F*	Alpha‐1F subunit of L‐type Ca^2+^ channel	Xp11.23	Modulates the biophysical properties of L‐type Ca^2+^ channel	Congenital night blindness and autism	Breitenkamp et al. (2015) and Hemara‐Wahanui et al. (2005)
*CACNA1G*	Alpha‐1G subunit of T‐type Ca^2+^ channel	17q21.33	Modulates the Ca^2+^ influx of T‐type channel in neurons and muscle	ASD, intellectual disability, Juvenile myoclonic epilepsy	Girirajan et al. (2013)
*CACNA1H*	Alpha‐1H subunit of T‐type Ca^2+^ channel	16p13.3	Abundantly expressed in cerebellum and cerebral cortex, activates small depolarization and contributes to the oscillatory behavior in neurons	ASD, childhood absence epilepsy, idiopathic generalized epilepsy	Splawski et al. (2006)
*CACNA1I*	Alpha‐1I subunit of T‐type Ca^2+^ channel	22q13.1	Modulates the Ca^2+^ influx of T‐type channel in neurons and generates pacemaker activity		Breitenkamp et al. (2015) and Hussman et al. (2011)
*CACNA2D4*	Alpha‐2/delta‐4 subunit of voltage‐gated Ca^2+^ channel	12p13.33	Modulates Ca^2+^ influx and voltage‐gated channel properties	Retinal cone dystrophy 4, ASD (when gene deletion occurs along with CACNA1C)	Smith et al. (2012)
*CACNA2D3*	Alpha‐2/delta‐3 subunit of voltage‐gated Ca^2+^ channel	3p21.1–p14.3	Modulates Ca^2+^ influx and voltage‐gated channel properties	ASD	Breitenkamp et al. (2015) and Skafidas et al. (2014)
*CACNB2*	Beta‐2 subunit of voltage‐gated Ca^2+^ channel	10p12.33–p12.31	Modulates the kinetics of L‐type calcium channel by increasing its activity	ASD, psychiatric diseases, Brugada syndrome	Breitenkamp et al. (2014)
*SCN1A*	Voltage‐regulated sodium channel type 1	2q24.3	Expressed in neurons and central and peripheral nervous system. Highly conserved through evolution. Controls channel gating and current	Inherited seizure disorder, Generalized Epilepsy with Febrile Seizures Plus (GEFS+), Juvenile myoclonic epilepsy, mental retardation, ASD	Craig et al. (2012), O'Roak et al. (2011), and Weiss et al. (2003)
*SCN2A*	Voltage‐regulated sodium channel type 2	2q24.3	Expressed in neurons and central and peripheral nervous system. Controls channel gating and current	Early infantile epileptic, encephalopathy, benign familial infantile seizures, ASD	Celle et al. (2013) and Weiss et al. (2003)
*SCN3A*	Voltage‐regulated sodium channel type 3	2q24.3	Expressed in neurons and central and peripheral nervous system. Controls biophysical properties of the channel	Epilepsy, ASD	Celle et al. (2013) and Weiss et al. (2003)
*SCN7A*	Voltage‐regulated sodium channel type 7	2q24.3	Na^+^‐specific channel in excitable cells	ASD (homozygous deletion in autism)	Morrow et al. (2008)
*SCN8A*	Voltage‐regulated sodium channel type 8	12q13.13	Alters the repetitive firing pattern of cerebellar Purkinje neurons	Cerebellar ataxia, epileptic encephalopathy early infantile, ASD	Weiss et al. (2003)
*KCNMA1*	Calcium‐activated large conductance potassium channel subfamily A	10q22.3	Synaptic protein regulator of neuronal excitability	Generalized epilepsy and paroxysmal dyskinesia (GEPD), ASD	Laumonnier et al. (2006)
*KCNMB4*	BK channel beta subunit 4	12q15	Regulatory subunit of BK channel	ASD	Skafidas et al. (2014)
*KCNQ3*	Potassium voltage‐gated channel (M‐channel)	8q24.22	Modulates the kinetics of the channel	Rolandic epilepsy and idiopathic generalized epilepsy (IGE) including benign neonatal convulsions, ASD	Gilling et al. (2013)
*KCNQ5*	Potassium voltage‐gated channel (M‐channel)	6q13	Expressed in brain and muscle and implicated in slow activation of the channel. Interacts with KCNQ3	ASD	Gilling et al. (2013)
*GRIK2*	Glutamate receptor ionotropic kainate 2	6q16.3	Glutamate receptors are the predominant excitatory neurotransmitter receptors in the central nervous system. Converts chemical signal to electrical impulse	Mental retardation, ASD	Ben‐Ari et al. (2012), Kang and Barnes (2013), and Laumonnier et al. (2006)
*GRIK3*	Glutamate receptor ionotropic kainate 3	1p34.3	Paralog of GRIK2	Schizophrenia, ASD	Ben‐Ari et al. (2012), Kang and Barnes, 2013, and Laumonnier et al. (2006)
*CHRNA7*	Acetylcholine receptor, neuronal nicotinic, alpha‐7 subunit	15q13.3	Postsynaptic GABAergic interneuron activity. Mediates fast signal transmission at synapses	Schizophrenia, ASD	Ben‐Ari et al. (2012), Kang and Barnes (2013), and Laumonnier et al. (2006)
*GABRG3*	GABA‐A gamma subunit of GABA receptor family	15q12	Conducts chloride ions upon activation leading to hyperpolarization. Causes inhibitory effect on neurotransmission	Schizophrenia, ASD	Ben‐Ari et al. (2012), Kang and Barnes, 2013, and Laumonnier et al. (2006)

The importance of defective regulation of intracellular Ca^2+^ in the pathophysiology of ASD is further supported by the association between genes that encode plasma membrane Ca^2+^ pumps and ASD. In fact, three studies from different human populations reported an association between the *ATP2B2* gene coding for the plasma membrane Ca^2+^ ATPase and ASD phenotypes (Yang et al., 2013). It should be noted that ASD‐associated genetic variations have been identified in genes encoding Ca^2+^ channels and Ca^2+^ transport pumps, as well as in genes encoding ion channels whose activities are under Ca^2+^ modulation. To the best of our knowledge, until now, no association with other Ca^2+^ channels such as ligand‐gated Ca^2+^ channels (RyR, IP3R) has been found, hampering the exploration of novel cellular pathways.

#### 2.2.2 Potassium (K^+^) channels in ASD

K^+^ channels are located in membranes of excitable and non‐excitable cells and they assure K efflux out of cells. According to their structure and functions, K^+^ channels are segregated into four categories: the voltage‐gated channels, inwardly rectifying (Kir), tandem pore domain (K2P), and the ligand‐gated (Kligand) channels (Kuang, Purhonen, & Hebert, 2015). They all share a pore‐forming α subunit but different regulatory subunits are identified in each group. Ca^2+^‐activated potassium channels (BK_Ca_) are ligand‐gated K^+^ channels that participate to several cell functions such as the regulation of hormone and neurotransmitter releases (Kuang et al., 2015). In fact, BK_Ca_ are abundantly distributed throughout the brain and are mainly localized at presynaptic terminals, where they partake in the adjustment of synaptic transmission and neuronal excitability (Kuang et al., 2015; Laumonnier et al., 2006). Laumonnier et al. (2006) observed a de novo balanced translocation of the 9q23/10q22 region that houses the α_1_ subunit gene of BK_Ca_ channel (*KCNMA1)* in patients with ASD. Electrophysiological experiments on lymphoblastoid cell lines derived from patients with ASD manifested a reduced activity of these channels. The authors also found a missense variation that alters a conserved domain of the channel in one patient with ASD (Laumonnier et al., 2006; Table 2). Furthermore, a variation in the α_1_ subunit of BK_Ca_ channel (*KCNMA1)* has been implicated in generalized epilepsy and paroxysmal dyskinesia (Du et al., 2005). A novel missense variation (c.595A.T) in *KCNB1* gene that encodes K_V_2.1 voltage‐gated potassium channel was detected in a patient with ASD associated with intellectual disability and epilepsy. This variation causes the substitution of isoleucine to phenylalanine at codon 199 (p.I199F) leading to significant depolarizing shifts in the voltage dependence of activation and inactivation of the channel (Calhoun, Vanoye, Kok, George, & Kearney, 2017; Table 2). The regulatory β_4_ subunit gene of BK_Ca_ channel (*KCNMB4*) was classified as one of the three predictive genes in ASD as it was strongly associated with SNPs‐ASD‐associated in a large meta‐analysis study (Skafidas et al., 2014; Table 3). On the other hand, a variation of *KCNQ3* gene mapped to chromosome 8q24, encoding the voltage‐gated potassium channel K_v_7.3, has been linked to epilepsy. This locus was found disrupted as a consequence of a de novo chromosomal translocation in one patient with ASD. In addition, three patients with ASD shared a missense variation in *KCNQ3*. This variation could be described as a loss of function as identified by electrophysiological recordings in *Xenopus laevis* oocytes (Gilling et al., 2013; Table 2).

These findings established a link between ASD and potassium channels and highlight their physiological importance in neuronal functions.

#### 2.2.3 Sodium (Na^+^) Channels in ASD

Voltage‐gated Na^+^ channels (Na_v_) are essential for the initiation and propagation of action potentials in neuronal cells, muscles, and heart tissues. Na_V_ channels are heteromeric complexes comprised of an α subunit (pore‐forming) associated with one or more β regulatory subunits. We distinguish nine members of Na_V_ channels (Na_V_1.1 to Na_V_1.9) that differ by their structure but also by their ligand‐specific binding sites (toxins, drugs) which has led to their classification as critical drug targets (Bagal, Marron, Owen, Storer, & Swain, 2015).

Na_v_ channels are primarily expressed in neurons and glial cells in the central and peripheral nervous system. Variations affecting the α subunit of Na^+^ channels and their accessory β subunit are known to be responsible for Brugada syndrome, a cardiac disease (Weiss et al., 2003). In addition, several variations in *SCN1A* and *SCN2A* that encode Na_v_1.1 and Na_v_1.2, respectively, are associated with childhood epilepsy and ASD (Weiss et al., 2003). Variations in *SCN1A* and *SCN2A* were shown to cause familial hemiplegic migraine and to be implicated in severe seizure syndrome, epilepsy, and Dravet syndrome (Craig, de Menezes, & Saneto, 2012; Weiss et al., 2003). It was shown that variations in *SCN2A* affect the calmodulin‐binding site of the channel and reduce its affinity for Ca^2+^. This site is crucial for the binding between channel subunits and for connecting Na^+^ channels to Ca^2+^ signaling pathways (Weiss et al., 2003). Another study using array‐comparative genome hybridization identified a de novo deletion in a chromosome 2 region (2q24.2–q24.3) that contains *SCN2A* and *SCN3A* genes in a child with ASD (Celle, Cuoco, Porta, Gimelli, & Tassano, 2013). These findings were also validated by whole‐exome sequencing study showing a significant association of *SCN1A* gene with the etiology of ASD (O'Roak et al., 2012; Sanders et al., 2012). Tavassoli et al. (2014) using whole‐exome sequencing found a de novo splice site variation in *SCN2A* gene in one patient with ASD. This variation (c.476+1G>A) that occurs at exon 4 of *SCNA2A* gene generates a truncated protein (Tavassoli et al., 2014; Table 2). In addition, the α subunit 8 gene of a Na^+^ channel was associated with ASD and identified by whole‐genome sequencing in a family with ASD. In fact, a de novo heterozygous missense variation was found in *SCN8A* gene (p.N1768D), which alters a conserved residue of the channel. The biophysical consequences of this variation are an increase in Na^+^ current and partial channel inactivation (Veeramah et al., 2012; Table 2). In a large study of consanguineous families with autism, a homozygous deletion of *SCN7A* gene was identified in one family, which is adjacent to *SCN1A* gene within the sodium channel gene cluster (*SCN1A*,* SCN2A*,* SCN3A*, and *SCN9A*) on chromosome 2 (Morrow et al., 2008; Table 3).

#### 2.2.4 Role of the ligand‐gated ion channels, GABA, glutamate, and cholinergic nicotinic receptors in ASD

Due to their crucial role in synaptic transmission, variations in GABA‐A receptors are implicated in several severe neurological and neuropsychiatric disorders (Kang & Barnes, 2013). Patients with ASD have been reported to carry rearrangements abnormalities in chromosome 15q11–13 known as the imprinted region of Angelman/Prader–Willi syndromes. This region houses a cluster of GABA receptor genes that include *GABRA5*,* GABRG3,* and *GABRB3,* as well as *CHRNA7* encoding the α7 subunit of the nicotinic acetylcholine receptor (nAChR; Hoppman‐Chaney, Wain, Seger, Superneau, & Hodge, 2013). Moreover, polymorphisms in *GABRA4* have also been associated with autism. Two SNPs located within 15q12 region were significantly linked to ASD, suggesting that this particular region of *GABRG3* gene is associated with an increased risk for ASD (Ben‐Ari, Khalilov, Kahle, & Cherubini, 2012; Kang & Barnes, 2013). ASD‐associated polymorphisms have been identified in both the *GRIK2* gene encoding the ionotropic glutamate receptor kainate 2 and the *GRM5* gene that encodes a metabotropic glutamate receptor. A recent study demonstrated a large spectrum of ASD phenotypes associated with the 15q11–13 microdeletions. This region includes the *CHRNA7* locus suggesting that *CHRNA7* is a critical gene in ASD (Kuang et al., 2015; Laumonnier et al., 2006).

GABA‐A receptors and Cl^−^ concentration are important actors for the excitation/inhibition balance in neurons during neurogenesis. These two actors appear to be complementary in that the level of Cl^−^ concentration is crucial for GABAergic signaling. The regulation of Cl^−^ concentration in neurons is mediated by Cl^−^ cotransporter (CCCs) proteins anchored into the plasma membrane. Their roles are to couple the transport of Na^+^, K^+^, and Cl^−^ and are named Na/K‐Cl (NCC, NKCC1, and NKCC2) transporters. There are four different K‐CCCs (KCC1, KCC2, KCC3, and KCC4). In neurons, NKCC1 and KCC2 are the predominant Cl^−^ exchangers (Ben‐Ari et al., 2012). A variation in *SLC12A2* gene encoding NKCC1 was reported to be linked to schizophrenia. Functional experiments in *Xenopus oocytes* of this variation displayed an increased sensitivity to the NKCC blocker bumetanide. This evidence supports the hypothesis that NKCC1 activity is associated with schizophrenia and ASD because these two conditions share the same genetic background (Merner et al., 2016). In fact, it was demonstrated that patients with ASD present an elevated intracellular Cl^−^ concentration in neurons, suggesting that defective excitability/inhibition balance could promote ASD due to an ineffective action of GABA leading to an abnormal chloride gradient (Ben‐Ari et al., 2012). Bumetanide blocks NKCC1 and decreases intracellular chloride concentration in neurons. A clinical study done on 60 children showed improvements in some ASD‐related clinical manifestations. Therefore, bumetanide is currently under investigation as a prospective drug by restoring the gradient and GABA inhibition and, thereby, considered as a potential ASD‐therapeutic agent (Lemonnier et al., 2012; Table 3).

### 2.3 Ion channels and dysfunctional pathways in ASD

Several genes encoding proteins involved in cellular pathways have been found enriched in ASD. These proteins are essentially implicated in synapse regulation (chromatin remolding, synaptic functions and protein synthesis and degradation; De Rubeis et al., 2014; Hormozdiari, Penn, Borenstein, & Eichler, 2015; Pinto et al., 2011; Ronemus, Iossifov, Levy, & Wigler, 2014; Uddin et al., 2014; Voineagu et al., 2011).

Synaptic regulatory proteins mainly concern: glutamatergic (e.g., GRIN2B) and GABAergic (e.g., GABRA3 and GABRB3) neurotransmission, neuronal connection (e.g., CNTNAP2) and ion permeability (e.g., CACNA1, CACNA2D3, and SCN1A), as well as proteins directly involved in synapse formation such as neurexins (NRXNs) and neuroligins (NLGNs). Among the scaffold proteins, there are proteins involved in the regulation of cell adhesion molecules and neurotransmitter receptors density in the synapse. This is the example of SHANK family proteins that assemble into large molecular platforms interacting with glutamate receptors, ion channels, actin cytoskeleton‐associated proteins, and G protein‐coupled signaling pathways (Grabrucker, Schmeisser, Schoen, & Boeckers, 2011). The SHANK proteins are associated with NMDA receptors via the guanylate kinase‐associated protein (GKAP)/postsynaptic density‐95 (PSD‐95) complex and with metabotropic glutamate receptors type 1 (mGluR1) *via* the neuronal scaffolding protein Homer1. In addition, SHANK proteins can bind to several actin‐regulatory molecules, such as cortactin (Durand et al., 2012). Mutations and CNVs (deletion and duplication) affecting *SHANK* genes have been associated with ASD. These variations resulted in actin accumulation in dendritic spines, which alters the development and the morphology of dendrites (Durand et al., 2012).

Furthermore, neuronal dysfunctions are due to the modifications in synthesis level of synaptic proteins caused by a defective mRNA regulation especially translation (Kelleher & Bear, 2008). This mechanism is controlled by several genes in particular *mTOR* and *FMR1*. FMRP protein, encoded by *FMR1* gene, binds to 400 different mRNAs and represses their translation (Kelleher & Bear, 2008). The loss of FMRP protein results in fragile X syndrome that is present in 5% in patients with ASD. This protein acts downstream of the Ras‐ERK signaling pathway *via* the complex FMRP–EIF4E–CYFIP1. This complex regulates the translation of more than 1,000 specific genes, many of which are ASD risk genes (De Rubeis et al., 2014). When CYFIP1 (cytoplasmic *FMR1* interacting protein 1) binds to FMRP protein, the complex inhibits directly the translation of mRNA or indirectly by preventing the ribosomal translocation on mRNA. It is interesting to note that the expression of FMRP is under the control of Ca^2+^/calmodulin‐dependent protein kinase 4 (CAMKIV). However, *CYFIP1* and *CAMKIV* have been described as susceptibility genes in ASD and together combined with an altered activity of FMPR enhances the ASD risk (Waltes et al., 2014).

Another pathway implicating calcium signaling and ASD is the Mammalian target of rapamycin (mTOR) pathway also known as the mechanistic target of rapamycin kinase. *MTOR* gene is a tumor suppressor that regulates calcium signaling and mitochondrial functions. mTOR controls cells growth, proliferation, and differentiation, involved in synapse plasticity, and inhibits autophagy by preventing protein degradation. Interestingly, mTOR is upstream regulated by several mediators such as growth factors signals (e.g., insulin) or in neurons by the brain‐derived neurotrophic factor (BDNF) through the phosphoinositide‐3‐kinase (PI3K) activation the protein kinase B (Akt) and Ras to the extracellular signal‐regulated kinase (Erk; Napoli et al., 2008; Schratt, Nigh, Chen, Hu, & Greenberg, 2004).

Both Erk and Akt act on the tuberous sclerosis complex (TSC1 and TSC2) by phosphorylating TSC2 inducing its dissociation of TSC1. TSC1 and TSC2 proteins act like GTPase proteins and downregulate a small GTPase Rheb (Ras homolog enriched in brain) protein *via* GAP protein through a mechanism that remains unknown (Ma & Blenis, 2009).

**Figure 1:**
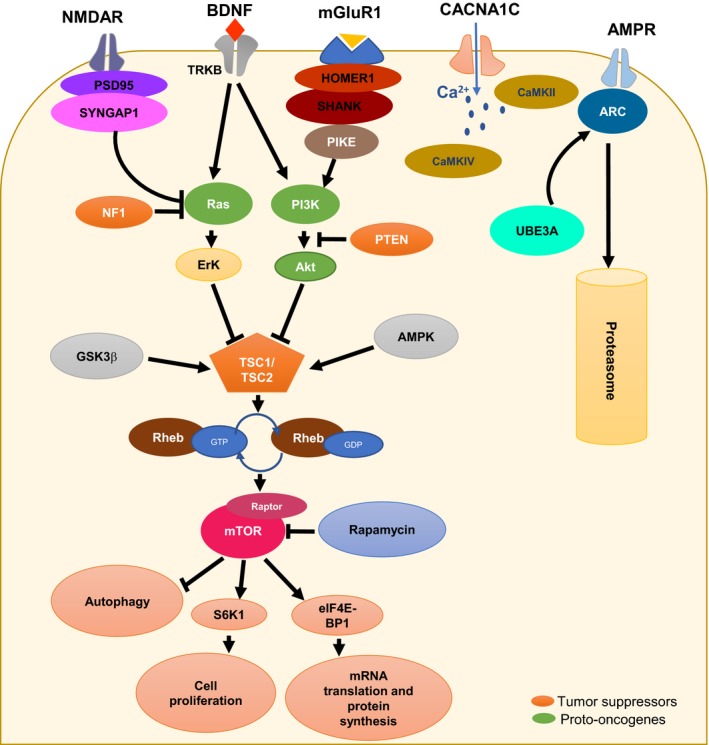
Synaptic signaling pathways associated with autism spectrum disorder (ASD). Alterations in the mechanistic target of rapamycin complex (mTOR) are considered risk factors for ASD. mTOR is activated by Rheb‐GTP. Upstream of Rheb is the tuberous sclerosis complex (TSC1–TSC2). TSC2 contains a GTPase‐activating protein (GAP) domain that converts Rheb from GTP‐bound form to its inactive GDP‐bound form. Several upstream signaling pathways ranging from PI3K–AKT, Ras–ERK, LKB1–AMPK and Wnt–GSK3β pathways, positively or negatively regulate mTOR signaling. (AMPK, AMP‐activated protein kinase; ERK, extracellular signal‐regulated kinase; GSK3β, glycogen synthase kinase 3β; and PI3K, phosphoinositide 3‐kinase). The mTOR pathway is also regulated by the brain‐derived neurotrophic factor (BDNF) which binds to the tropomyosin‐related kinase B (TRKB). BDNF plays a key role in the development and the plasticity of the central nervous system and it is considered a risk factor for ASD because increased levels of BDNF concentration have been observed in the serum and brain of patients with ASD. PI3K is also regulated by the synaptic protein SHANK, which is associated with metabotropic glutamate receptors type 1 (mGluR1) via the neuronal scaffolding protein HOMER1. The mTOR complex is a key modulator of protein synthesis by direct phosphorylation of 4E‐binding proteins (4E‐BPs) and activation of the ribosomal subunit S6 kinase (S6Ks), which in turn phosphorylate translation initiation factors. Thus, mTOR blocks the activation of cell autophagy and promotes cell proliferation, growth, and differentiation. The activity of the proteasome is also regulated by neuronal activity. The expression of UBE3A is increased through the transcription factor MEF2 and regulates the degradation of ARC protein, which promotes the internalization of AMPA‐R and regulates excitatory synapse development. Variations in the neuronal L‐type Ca^2+^ channel α subunit CACNA1C have been associated with Timothy syndrome and with ASD. In addition, Ca^2+^/calmodulin‐dependent protein kinases are associated with components of the neuronal complex including the fragile X mental retardation protein (FMRP) and its protein interaction CYFIP1, which also consider candidate genes in ASD. UBE3A: ubiquitin–protein ligase E3A; MEF2: myocyte‐specific enhancer factor 2; ARC: activity‐regulated cytoskeleton‐associated protein; AMPR: AMPA receptors; CYFIP1: cytoplasmic FMRP‐interacting protein 1

Rheb is a direct activator of mTOR complex by activating its regulatory associated protein (raptor; Ma & Blenis, 2009). Once the mTOR complex is activated, it phosphorylates a series of protein such as the S6 kinase 1 (S6K1), the eukaryotic translation initiation factor 4E‐binding protein 1 (eIF‐4BP1), and the carbamoyl‐phosphate synthetase 2, aspartate transcarbamylase, and dihydroorotase (CAD). S6K1 and eIF‐4BP1 are essential for protein synthesis and polypeptide translation in ribosomes and cell proliferation, while CAD is a key player in pyrimidine synthesis and so nucleotide synthesis (Ma & Blenis, 2009). In addition, the tuberous sclerosis complex can also be activated by AMPK, GSK3β, and p53 which leads the inhibition of mTOR pathway. Furthermore, variations in *TSC1* and *TSC2* genes have been associated with ASD (Devlin & Scherer, 2012). Also, variations in mTOR pathway repressors, such as for the neurofibromin 1 (NF1) gene *NF1*, cause neurofibromatosis type 1 syndrome as reported in 1% patients with ASD (Devlin & Scherer, 2012). The phosphatase and tension protein homolog (PTEN) is also known to downregulate mTOR pathway *via* both PI3K and AKT. Patients with ASD associated with cerebral malformation, like macrocephaly, have been found to carry variations in the *PTEN* gene in 7% of the cases (Devlin & Scherer, 2012; McBride et al., 2010; Figure 1).

Besides protein synthesis and translation that have been shown to be implicated in the process of ASD, the mechanism of protein degradation was also studied in ASD. Genetic studies indicate that ubiquitin–proteasome system is necessary for normal human cognitive function by regulating the synapse assembly and elimination (Mabb & Ehlers, 2010). The ubiquitin ligase enzyme Ube3A is a member of the E3 ubiquitin ligase family. The disruption of its activity leads to Angelman syndrome, while in turn the Angelman syndrome was described in ASD with CNVs and mutations in *UBE3A* gene (Greer et al., 2010). In Ube3A knockout mice, electrophysiological studies demonstrated an impaired long‐term potentiation (LTP) in the hippocampus, which suggest that alteration of Ube3A results in the loss of neuronal plasticity. In fact, Ube3A increases transcription through the myocyte enhancer factor 2 (MEF2) complex and regulates synapse function by ubiquitinating and degrading the synaptic protein Arc (activity‐regulated cytoskeleton‐associated protein). The role of Arc is to decrease long‐term potentiation by promoting the internalization of AMPA receptors, which are the mediators of the excitatory neurotransmission in the central nervous system (Greer et al., 2010). On another hand, a decrease in AMPAR expression at synapses has been observed in patients with fragile X syndrome. This decrease is due to excessive mGluR5 signaling resulting in an increased Arc translation and consequently excessive AMPA receptors internalization (Dolen & Bear, 2008). In FMR1 knockout mice, injections of mGluR5 restore the AMPA receptors expression levels and prevent fragile X syndrome (Dolen et al., 2007).

Interestingly, it has been shown that an alteration of the inhibitory phosphorylation function of the Ca^2+^/calmodulin‐dependent protein kinase II (CamKII) is coupled to an increase in AMPA receptors expressed at the synapse (Rose, Jin, & Craig, 2009). In addition, mutations affecting this critical site of CamKII were shown to prevent the behavioral deficit in *UBE3A* gene‐altered mice, suggesting that the Angelman syndrome is associated with a perturbation of CamKII functions (van Woerden et al., 2007; Figure 1).

Together, these studies emphasize the implication of ion channels in the pathophysiology of ASD and strengthen the hypothesis that pharmacological manipulation of ion channels function is a potential therapeutic target in ASD.

### 2.4 Ion channels and drug therapy in ASD

Ion channels have always been considered as powerful drug targets for the treatment of a wide range of pathologies owing to their crucial role as regulators of cell excitability (Kaczorowski, McManus, Priest, & Garcia, 2008).

In 1884, cocaine was discovered as the first anesthetic drug (Vandam, 1987). Several decades later, cocaine was described as a Na^+^ channel blocker (Kyle & Ilyin, 2007; Vandam, 1987). This observation led the chemists to the production of novel analogs of cocaine, all classified under the term of “caine” and constituting a novel family of anesthetics (e.g., benzocaine, lidocaine; Casale, Symeonidou, & Bartolo, 2017; Tremont‐Lukats, Megeff, & Backonja, 2000). Thereafter, drug‐mediated modulation of Na^+^ channel properties was found to have other therapeutic functions such as anticonvulsants and antidepressants (e.g., carbamazepine) used in the treatment of neuropathic pain (Tremont‐Lukats et al., 2000).

Valproic acid (VPA) is one of the most widely used anti‐epileptic drugs for the treatment of tonico‐clonic seizures that act by modulating Na^+^ channel kinetics in neurons (Loscher, 2002). VPA is also used for the treatment of bipolar disorder, anxiety, and migraine (Loscher, 2002). Studies showed that the exposure to VPA during pregnancy induces neurobehavioral abnormalities similar to autism traits in both rodents and humans (Bertelsen et al., 2016; Choi et al., 2016; Mony, Lee, Dreyfus, DiCicco‐Bloom, & Lee, 2016). In fact, VPA treatment of postnatal rats was shown to affect DNA synthesis and astrocyte proliferation and was associated with autistic behavior (Mony et al., 2016). A recent study showed that the phenotypic signs of ASD induced by VAP exposure in rats can be significantly improved or recovered by the administration of vitamin D in early stages of development (Du, Zhao, Duan, & Li, 2017). In addition, it has been demonstrated that persistent Na^+^ current is responsible for hypoxia in neurons leading to neuronal damages (Faustino & Donnelly, 2006). In fact, the persistence of Na^+^ currents leads to the increased activity of Na^+^/Ca^2+^ exchangers in neurons, itself resulting in an increase in Ca^2+^ cytoplasmic concentration (Faustino & Donnelly, 2006). In order to correct this situation, it has been proposed that an increase in Na^+^ influx into cells prevents trauma in the nervous system (Ates et al., 2007). Some Na^+^ channel blockers (e.g., phenytoin, riluzole) showed neuroprotective activity in experimental spinal cord injury studies, in neurobehavioral studies and tissue recovery (Ates et al., 2007). It is important to mention that gabapentin, the first known drug in the treatment of neuropathic pain, specifically binds to the α2δ1 subunit of N‐type Ca^2+^ channels and decreases the current (Zhu et al., 2017).

*N*‐methyl‐d‐aspartate receptors (NMDAR) are well known to be associated with psychiatric disorders (Lakhan, Caro, & Hadzimichalis, 2013). With no surprise, they were also linked to ASD risk (Lee, Choi, & Kim, 2015). Their activation follows the binding of glutamate once the D‐serine or glycine co‐agonists engage the specific allosteric site of the receptor (Kim et al., 2005). These two ligands were used in a clinical study on patients with severe schizophrenia as antipsychotic agents and were able to correct some negative clinical aspects (Buchanan et al., 2007). In 1991, Haring et al. characterized an antibody, named B6B21, which showed a remarkable action in rat neurons by increasing long‐term potentiation in CA1 pyramidal cells. This antibody has a high binding affinity for NMDA receptors. As a matter of fact, the authors concluded that B6B21 acts in a similar way to glycine on the receptor (Haring, Stanton, Scheideler, & Moskal, 1991). From B6B21, derived a family of small peptides called glyxines (Santini et al., 2014). One of these peptides, named GLYX‐13, was found to modulate NMDAR properties in a similar way to glycine. Treating ASD‐affected rats with of GLYX‐13 resulted in promising improvements of autistic signs. Thereafter, authors suggested that this antibody might be a potential treatment for patients affected by ASD (Santini et al., 2014). Moreover, d‐cycloserine, which is a partial NMDAR glycine agonist, is known to have effects on the behavioral deficits observed in autism and schizophrenia (Posey et al., 2004).

In a recent clinical trial carried out on 20 patients with autism, it has been shown that D‐cycloserine treatment alleviated the stereotyped behavior of these patients (Urbano et al., 2014). To go more into details, the administration of D‐cycloserine during 8 weeks with different dosages showed to be effective on ASD manifestations in these patients without showing any side effects (Urbano et al., 2014). Additional studies will be required to determine the therapeutic effect of this drug in ASD.

Concerning another therapeutic target, the implication of the acetylcholine receptor in ASD was demonstrated for the first time by the analysis of postmortem adult brains from patients that suffered ASD (Martin‐Ruiz et al., 2004). Analysis of mRNA levels by real‐time PCR in different brain tissues (cerebral cortex and cerebellum) showed a significant difference in the mRNA expression of several nicotinic acetylcholine receptor subunits (α3, α4, β2, and α7; Martin‐Ruiz et al., 2004). Thus, it was suggested that the loss of nAChR functionality in the brain could be responsible for the ASD phenotype (Martin‐Ruiz et al., 2004). Administration of acetylcholine receptor activator donepezil to an ASD‐affected boy proved beneficial for his cognitive skills after 6 weeks of treatment (Srivastava, Agarwal, & Pundhir, 2011). A randomized double‐blind placebo‐controlled trial using glutamine in autistic children showed significant improvements for some ASD clinical signs (Ghaleiha et al., 2014). The α7 nicotinic acetylcholine receptor encoded by the *CHRNA7* gene has also been associated with ASD (Deutsch, Urbano, Burket, Herndon, & Winebarger, 2011; Dineley, Pandya, & Yakel, 2015). Due to their role and implication in several pathways (e.g., PI3K/Akt and Wnt), α7 nAChR is considered as powerful therapeutic candidates (Deutsch, Burket, Urbano, & Benson, 2015).

## 3 CONCLUSIONS

A number of genetic studies came to classify autism as the most genetically complex disease. However, only a few numbers of contributing alleles or co‐inherited alleles are found in ASD proving that additional epigenetic factors or environmental conditions may contribute to the clinical manifestation of this disorder. The several Mendelian pathologies associated with autism, for example, fragile X syndrome, provide the strongest argument highlighting the genetic basis of autism. These ASD‐associated pathologies are commonly confounded with autistic behaviors and therefore make it more difficult to carry out case studies that focus exclusively on ASD. The most promising genes identified so far include *NLGN*,* SHANK,* and *SYNGAP1*, which are involved in neurogenesis and synaptogenesis, suggesting that synaptic malfunction is a significant contributor to the etiology of ASD. Among ASD‐associated pathologies, Timothy syndrome linked channelopathies to ASD. In fact, recent studies implicate variations and mutations of genes encoding ion channels (Ca^2+^, K^+^, Na^+^, and Cl^−^ channels) as a leading risk factor for ASD. Alterations of these channels highlight the complexity of the pathology that remains not fully understood. Interestingly, the wide implication of ion channels encoding genes in ASD may provide opportunities for pharmacological treatments of autistic patients because these channels represent powerful drug target. For instance, bumetanide is one of these potential therapeutic agents currently under evaluation. Some of ASD pathological conditions show an increase or decrease in ion channel activation/deactivation kinetics, suggesting that ion channel modulators may be therapeutic candidates for the treatment of ASD.

In conclusion, ASD is not a simple pathology and it is associated with a large spectrum of other diseases. Nevertheless, the genetic abnormalities so far indicate that defective neuronal function from the onset of neural development appears to be a leading cause in the manifestation of this syndrome.

Furthermore, defective regulation of ion flux through the cell membrane caused by altered kinetics of ion channels and transporters appears to cause an imbalance of excitation/inhibition in neural function that may lead to defective neuronal circuit formation and physiological response. Restoring ion dynamics to their physiological equilibrium may represent a promising therapeutic strategy for this devastating neurodevelopmental psychiatric disorder.

## CONFLICT OF INTEREST

All authors state that they have no conflict of interests.

## References

[brb3978-bib-0001] American Psychiatric Association (2013). Diagnostic and statistical manual of mental disorders fifth edition (DSM‐5) (pp. 31). Washington, DC: American Psychiatric Publisher.

[brb3978-bib-0002] Anney, R. , Klei, L. , Pinto, D. , Regan, R. , Conroy, J. , Magalhaes, T. R. , … Hallmayer, J. (2010). A genome‐wide scan for common alleles affecting risk for autism. Human Molecular Genetics, 19(20), 4072–4082. 10.1093/hmg/ddq307 20663923 PMC2947401

[brb3978-bib-0003] Ates, O. , Cayli, S. R. , Gurses, I. , Turkoz, Y. , Tarim, O. , Cakir, C. O. , & Kocak, A. (2007). Comparative neuroprotective effect of sodium channel blockers after experimental spinal cord injury. Journal of Clinical Neuroscience, 14(7), 658–665. 10.1016/j.jocn.2006.03.023 17532502

[brb3978-bib-0004] Badescu, G. M. , Filfan, M. , Sandu, R. E. , Surugiu, R. , Ciobanu, O. , & Popa‐Wagner, A. (2016). Molecular mechanisms underlying neurodevelopmental disorders, ADHD and autism. Romanian Journal of Morphology and Embryology, 57(2), 361–366. 27516006

[brb3978-bib-0005] Bagal, S. K. , Marron, B. E. , Owen, R. M. , Storer, R. I. , & Swain, N. A. (2015). Voltage gated sodium channels as drug discovery targets. Channels (Austin), 9(6), 360–366. 10.1080/19336950.2015.1079674 26646477 PMC4850042

[brb3978-bib-0006] Barrett, C. F. , & Tsien, R. W. (2008). The Timothy syndrome mutation differentially affects voltage‐ and calcium‐dependent inactivation of CaV1.2L‐type calcium channels. Proceedings of the National Academy of Sciences of the United States of America, 105(6), 2157–2162. 10.1073/pnas.0710501105 18250309 PMC2538892

[brb3978-bib-0007] Ben‐Ari, Y. , Khalilov, I. , Kahle, K. T. , & Cherubini, E. (2012). The GABA excitatory/inhibitory shift in brain maturation and neurological disorders. The Neuroscientist, 18(5), 467–486. 10.1177/1073858412438697 22547529

[brb3978-bib-0008] Bertelsen, F. , Moller, A. , Folloni, D. , Drasbek, K. R. , Scheel‐Kruger, J. , & Landau, A. M. (2016). Increased GABAA receptor binding in amygdala after prenatal administration of valproic acid to rats. Acta Neuropsychiatrica, 29, 309–314. 27938419 10.1017/neu.2016.59

[brb3978-bib-0009] Bidaud, I. , Mezghrani, A. , Swayne, L. A. , Monteil, A. , & Lory, P. (2006). Voltage‐gated calcium channels in genetic diseases. Biochimica et Biophysica Acta, 1763(11), 1169–1174. 10.1016/j.bbamcr.2006.08.049 17034879

[brb3978-bib-0010] Boczek, N. J. , Miller, E. M. , Ye, D. , Nesterenko, V. V. , Tester, D. J. , Antzelevitch, C. , … Ware, S. M. (2015). Novel Timothy syndrome mutation leading to increase in CACNA1C window current. Heart Rhythm, 12(1), 211–219. 10.1016/j.hrthm.2014.09.051 25260352 PMC4907369

[brb3978-bib-0011] Bonora, E. , Graziano, C. , Minopoli, F. , Bacchelli, E. , Magini, P. , Diquigiovanni, C. , … Romeo, G. (2014). Maternally inherited genetic variants of CADPS2 are present in autism spectrum disorders and intellectual disability patients. EMBO Molecular Medicine, 6(6), 795–809. 24737869 10.1002/emmm.201303235 PMC4203356

[brb3978-bib-0012] Bourgeron, T. (2015). From the genetic architecture to synaptic plasticity in autism spectrum disorder. Nature Reviews Neuroscience, 16(9), 551–563. 10.1038/nrn3992 26289574

[brb3978-bib-0013] Breitenkamp, A. F. , Matthes, J. , & Herzig, S. (2015). Voltage‐gated calcium channels and autism spectrum disorders. Current Molecular Pharmacology, 8(2), 123–132. 10.2174/1874467208666150507105235 25966693

[brb3978-bib-0014] Breitenkamp, A. F. , Matthes, J. , Nass, R. D. , Sinzig, J. , Lehmkuhl, G. , Nurnberg, P. , & Herzig, S. (2014). Rare mutations of CACNB2 found in autism spectrum disease‐affected families alter calcium channel function. PLoS One, 9(4), e95579 10.1371/journal.pone.0095579 24752249 PMC3994086

[brb3978-bib-0015] Buchanan, R. W. , Javitt, D. C. , Marder, S. R. , Schooler, N. R. , Gold, J. M. , McMahon, R. P. , … Carpenter, W. T. (2007). The Cognitive and Negative Symptoms in Schizophrenia Trial (CONSIST): The efficacy of glutamatergic agents for negative symptoms and cognitive impairments. The American Journal of Psychiatry, 164(10), 1593–1602. 10.1176/appi.ajp.2007.06081358 17898352

[brb3978-bib-0016] Calhoun, J. D. , Vanoye, C. G. , Kok, F. , George Jr, A. L. , & Kearney, J. A. (2017). Characterization of a KCNB1 variant associated with autism, intellectual disability, and epilepsy. Neurology Genetics, 3(6), e198 10.1212/NXG.0000000000000198 29264390 PMC5733249

[brb3978-bib-0017] Casale, R. , Symeonidou, Z. , & Bartolo, M. (2017). Topical treatments for localized neuropathic pain. Current Pain and Headache Reports, 21(3), 15 10.1007/s11916-017-0615-y 28271334 PMC5340828

[brb3978-bib-0018] Celle, M. E. , Cuoco, C. , Porta, S. , Gimelli, G. , & Tassano, E. (2013). Interstitial 2q24.3 deletion including SCN2A and SCN3A genes in a patient with autistic features, psychomotor delay, microcephaly and no history of seizures. Gene, 532(2), 294–296. 10.1016/j.gene.2013.09.073 24080482

[brb3978-bib-0019] Choi, C. S. , Gonzales, E. L. , Kim, K. C. , Yang, S. M. , Kim, J. W. , Mabunga, D. F. , … Shin, C. Y. (2016). The transgenerational inheritance of autism‐like phenotypes in mice exposed to valproic acid during pregnancy. Scientific Reports, 6, 36250 10.1038/srep36250 27819277 PMC5098241

[brb3978-bib-0020] Cotney, J. , Muhle, R. A. , Sanders, S. J. , Liu, L. , Willsey, A. J. , Niu, W. , … Noonan, J. P. (2015). The autism‐associated chromatin modifier CHD8 regulates other autism risk genes during human neurodevelopment. Nature Communications, 6, 6404 10.1038/ncomms7404 PMC4355952 25752243

[brb3978-bib-0021] Craig, A. K. , de Menezes, M. S. , & Saneto, R. P. (2012). Dravet syndrome: Patients with co‐morbid SCN1A gene mutations and mitochondrial electron transport chain defects. Seizure, 21(1), 17–20. 10.1016/j.seizure.2011.08.010 21906962

[brb3978-bib-0022] Cross‐Disorder Group of the Psychiatric Genomics Consortium (2013). Identification of risk loci with shared effects on five major psychiatric disorders: A genome‐wide analysis. Lancet, 381(9875), 1371–1379. 23453885 10.1016/S0140-6736(12)62129-1 PMC3714010

[brb3978-bib-0023] De Rubeis, S. , He, X. , Goldberg, A. P. , Poultney, C. S. , Samocha, K. , Cicek, A. E. , … Buxbaum, J. D. (2014). Synaptic, transcriptional and chromatin genes disrupted in autism. Nature, 515(7526), 209–215. 10.1038/nature13772 25363760 PMC4402723

[brb3978-bib-0024] Deutsch, S. I. , Burket, J. A. , Urbano, M. R. , & Benson, A. D. (2015). The alpha7 nicotinic acetylcholine receptor: A mediator of pathogenesis and therapeutic target in autism spectrum disorders and Down syndrome. Biochemical Pharmacology, 97(4), 363–377. 10.1016/j.bcp.2015.06.005 26074265

[brb3978-bib-0025] Deutsch, S. I. , Urbano, M. R. , Burket, J. A. , Herndon, A. L. , & Winebarger, E. E. (2011). Pharmacotherapeutic implications of the association between genomic instability at chromosome 15q13.3 and autism spectrum disorders. Clinical Neuropharmacology, 34(6), 203–205. 10.1097/WNF.0b013e31823a1247 22094647

[brb3978-bib-0026] Devlin, B. , & Scherer, S. W. (2012). Genetic architecture in autism spectrum disorder. Current Opinion in Genetics & Development, 22(3), 229–237. 10.1016/j.gde.2012.03.002 22463983

[brb3978-bib-0027] Dineley, K. T. , Pandya, A. A. , & Yakel, J. L. (2015). Nicotinic ACh receptors as therapeutic targets in CNS disorders. Trends in Pharmacological Sciences, 36(2), 96–108. 10.1016/j.tips.2014.12.002 25639674 PMC4324614

[brb3978-bib-0028] Dolen, G. , & Bear, M. F. (2008). Role for metabotropic glutamate receptor 5 (mGluR5) in the pathogenesis of fragile X syndrome. The Journal of Physiology, 586(6), 1503–1508. 10.1113/jphysiol.2008.150722 18202092 PMC2375688

[brb3978-bib-0029] Dolen, G. , Osterweil, E. , Rao, B. S. , Smith, G. B. , Auerbach, B. D. , Chattarji, S. , & Bear, M. F. (2007). Correction of fragile X syndrome in mice. Neuron, 56(6), 955–962. 10.1016/j.neuron.2007.12.001 18093519 PMC2199268

[brb3978-bib-0030] Du, W. , Bautista, J. F. , Yang, H. , Diez‐Sampedro, A. , You, S. A. , Wang, L. , … Wang, Q. K. (2005). Calcium‐sensitive potassium channelopathy in human epilepsy and paroxysmal movement disorder. Nature Genetics, 37(7), 733–738. 10.1038/ng1585 15937479

[brb3978-bib-0031] Du, L. , Zhao, G. , Duan, Z. , & Li, F. (2017). Behavioral improvements in a valproic acid rat model of autism following vitamin D supplementation. Psychiatry Research, 253, 28–32. 10.1016/j.psychres.2017.03.003 28324861

[brb3978-bib-0032] Durand, C. M. , Betancur, C. , Boeckers, T. M. , Bockmann, J. , Chaste, P. , Fauchereau, F. , … Bourgeron, T. (2007). Mutations in the gene encoding the synaptic scaffolding protein SHANK3 are associated with autism spectrum disorders. Nature Genetics, 39(1), 25–27. 10.1038/ng1933 17173049 PMC2082049

[brb3978-bib-0033] Durand, C. M. , Perroy, J. , Loll, F. , Perrais, D. , Fagni, L. , Bourgeron, T. , … Sans, N. (2012). SHANK3 mutations identified in autism lead to modification of dendritic spine morphology via an actin‐dependent mechanism. Molecular Psychiatry, 17(1), 71–84. 10.1038/mp.2011.57 21606927 PMC3252613

[brb3978-bib-0034] Faustino, E. V. , & Donnelly, D. F. (2006). An important functional role of persistent Na^+^ current in carotid body hypoxia transduction. Journal of Applied Physiology, 101(4), 1076–1084. 10.1152/japplphysiol.00090.2006 16778007

[brb3978-bib-0035] Ghaleiha, A. , Ghyasvand, M. , Mohammadi, M. R. , Farokhnia, M. , Yadegari, N. , Tabrizi, M. , & Akhondzadeh, S. (2014). Galantamine efficacy and tolerability as an augmentative therapy in autistic children: A randomized, double‐blind, placebo‐controlled trial. Journal of Psychopharmacology, 28(7), 677–685. 10.1177/0269881113508830 24132248

[brb3978-bib-0036] Gilling, M. , Rasmussen, H. B. , Calloe, K. , Sequeira, A. F. , Baretto, M. , Oliveira, G. , … Tommerup, N. (2013). Dysfunction of the heteromeric KV7.3/KV7.5 potassium channel is associated with autism spectrum disorders. Frontiers in Genetics, 4, 54. 23596459 10.3389/fgene.2013.00054 PMC3627139

[brb3978-bib-0037] Girirajan, S. , Dennis, M. Y. , Baker, C. , Malig, M. , Coe, B. P. , Campbell, C. D. , … Eichler, E. E. (2013). Refinement and discovery of new hotspots of copy‐number variation associated with autism spectrum disorder. American Journal of Human Genetics, 92(2), 221–237. 10.1016/j.ajhg.2012.12.016 23375656 PMC3567267

[brb3978-bib-0038] Grabrucker, A. M. , Schmeisser, M. J. , Schoen, M. , & Boeckers, T. M. (2011). Postsynaptic ProSAP/Shank scaffolds in the cross‐hair of synaptopathies. Trends in Cell Biology, 21(10), 594–603. 10.1016/j.tcb.2011.07.003 21840719

[brb3978-bib-0039] Greer, P. L. , Hanayama, R. , Bloodgood, B. L. , Mardinly, A. R. , Lipton, D. M. , Flavell, S. W. , & Greenberg, M. E. (2010). The Angelman Syndrome protein Ube3A regulates synapse development by ubiquitinating arc. Cell, 140(5), 704–716. 10.1016/j.cell.2010.01.026 20211139 PMC2843143

[brb3978-bib-0040] Haring, R. , Stanton, P. K. , Scheideler, M. A. , & Moskal, J. R. (1991). Glycine‐like modulation of N‐methyl‐D‐aspartate receptors by a monoclonal antibody that enhances long‐term potentiation. Journal of Neurochemistry, 57(1), 323–332. 10.1111/j.1471-4159.1991.tb02131.x 1828831

[brb3978-bib-0041] Hemara‐Wahanui, A. , Berjukow, S. , Hope, C. I. , Dearden, P. K. , Wu, S. B. , Wilson‐Wheeler, J. , … Maw, M. A. (2005). A CACNA1F mutation identified in an X‐linked retinal disorder shifts the voltage dependence of Cav1.4 channel activation. Proceedings of the National Academy of Sciences of the United States of America, 102(21), 7553–7558. 10.1073/pnas.0501907102 15897456 PMC1140436

[brb3978-bib-0042] Heyes, S. , Pratt, W. S. , Rees, E. , Dahimene, S. , Ferron, L. , Owen, M. J. , & Dolphin, A. C. (2015). Genetic disruption of voltage‐gated calcium channels in psychiatric and neurological disorders. Progress in Neurobiology, 134, 36–54. 10.1016/j.pneurobio.2015.09.002 26386135 PMC4658333

[brb3978-bib-0043] Hoppman‐Chaney, N. , Wain, K. , Seger, P. R. , Superneau, D. W. , & Hodge, J. C. (2013). Identification of single gene deletions at 15q13.3: Further evidence that CHRNA7 causes the 15q13.3 microdeletion syndrome phenotype. Clinical Genetics, 83(4), 345–351. 10.1111/j.1399-0004.2012.01925.x 22775350

[brb3978-bib-0044] Hormozdiari, F. , Penn, O. , Borenstein, E. , & Eichler, E. E. (2015). The discovery of integrated gene networks for autism and related disorders. Genome Research, 25(1), 142–154. 10.1101/gr.178855.114 25378250 PMC4317170

[brb3978-bib-0045] Hussman, J. P. , Chung, R. H. , Griswold, A. J. , Jaworski, J. M. , Salyakina, D. , Ma, D. , … Pericak‐Vance, M. A. (2011). A noise‐reduction GWAS analysis implicates altered regulation of neurite outgrowth and guidance in autism. Molecular Autism, 2(1), 1 10.1186/2040-2392-2-1 21247446 PMC3035032

[brb3978-bib-0046] Jacquemont, M. L. , Sanlaville, D. , Redon, R. , Raoul, O. , Cormier‐Daire, V. , Lyonnet, S. , … Philippe, A. (2006). Array‐based comparative genomic hybridisation identifies high frequency of cryptic chromosomal rearrangements in patients with syndromic autism spectrum disorders. Journal of Medical Genetics, 43(11), 843–849. 10.1136/jmg.2006.043166 16840569 PMC2563185

[brb3978-bib-0047] Jamain, S. , Quach, H. , Betancur, C. , Rastam, M. , Colineaux, C. , Gillberg, I. C. , … Paris Autism Research International Sibpair Study (2003). Mutations of the X‐linked genes encoding neuroligins NLGN3 and NLGN4 are associated with autism. Nature Genetics, 34(1), 27–29. 10.1038/ng1136 12669065 PMC1925054

[brb3978-bib-0048] Kaczorowski, G. J. , McManus, O. B. , Priest, B. T. , & Garcia, M. L. (2008). Ion channels as drug targets: The next GPCRs. The Journal of General Physiology, 131(5), 399–405. 10.1085/jgp.200709946 18411331 PMC2346569

[brb3978-bib-0049] Kalkman, H. O. (2012). A review of the evidence for the canonical Wnt pathway in autism spectrum disorders. Molecular Autism, 3(1), 10 10.1186/2040-2392-3-10 23083465 PMC3492093

[brb3978-bib-0050] Kang, J. Q. , & Barnes, G. (2013). A common susceptibility factor of both autism and epilepsy: Functional deficiency of GABA A receptors. Journal of Autism and Developmental Disorders, 43(1), 68–79. 10.1007/s10803-012-1543-7 22555366

[brb3978-bib-0051] Kelleher 3rd, R. J. , & Bear, M. F. (2008). The autistic neuron: Troubled translation? Cell, 135(3), 401–406. 10.1016/j.cell.2008.10.017 18984149

[brb3978-bib-0052] Kim, P. M. , Aizawa, H. , Kim, P. S. , Huang, A. S. , Wickramasinghe, S. R. , Kashani, A. H. , … Snyder, S. H. (2005). Serine racemase: Activation by glutamate neurotransmission via glutamate receptor interacting protein and mediation of neuronal migration. Proceedings of the National Academy of Sciences of the United States of America, 102(6), 2105–2110. 10.1073/pnas.0409723102 15684087 PMC548584

[brb3978-bib-0053] Ko, C. , Kim, N. , Kim, E. , Song, D. H. , & Cheon, K. A. (2016). The effect of epilepsy on autistic symptom severity assessed by the social responsiveness scale in children with autism spectrum disorder. Behavioral and Brain Functions, 12(1), 20 10.1186/s12993-016-0105-0 27350381 PMC4924297

[brb3978-bib-0054] Krey, J. F. , & Dolmetsch, R. E. (2007). Molecular mechanisms of autism: A possible role for Ca^2+^ signaling. Current Opinion in Neurobiology, 17(1), 112–119. 10.1016/j.conb.2007.01.010 17275285

[brb3978-bib-0055] Krumm, N. , O'Roak, B. J. , Shendure, J. , & Eichler, E. E. (2014). A de novo convergence of autism genetics and molecular neuroscience. Trends in Neurosciences, 37(2), 95–105. 10.1016/j.tins.2013.11.005 24387789 PMC4077788

[brb3978-bib-0056] Kuang, Q. , Purhonen, P. , & Hebert, H. (2015). Structure of potassium channels. Cellular and Molecular Life Sciences, 72(19), 3677–3693. 10.1007/s00018-015-1948-5 26070303 PMC4565861

[brb3978-bib-0057] Kyle, D. J. , & Ilyin, V. I. (2007). Sodium channel blockers. Journal of Medicinal Chemistry, 50(11), 2583–2588. 10.1021/jm061005v 17489575

[brb3978-bib-0058] La Malfa, G. , Lassi, S. , Bertelli, M. , Salvini, R. , & Placidi, G. F. (2004). Autism and intellectual disability: A study of prevalence on a sample of the Italian population. Journal of Intellectual Disability Research, 48(3), 262–267. 10.1111/j.1365-2788.2003.00567.x 15025669

[brb3978-bib-0059] Lai, M. C. , Lombardo, M. V. , Auyeung, B. , Chakrabarti, B. , & Baron‐Cohen, S. (2015). Sex/gender differences and autism: Setting the scene for future research. Journal of the American Academy of Child and Adolescent Psychiatry, 54(1), 11–24. 10.1016/j.jaac.2014.10.003 25524786 PMC4284309

[brb3978-bib-0060] Lakhan, S. E. , Caro, M. , & Hadzimichalis, N. (2013). NMDA receptor activity in neuropsychiatric disorders. Frontiers in Psychiatry, 4, 52. 23772215 10.3389/fpsyt.2013.00052 PMC3677126

[brb3978-bib-0061] Laumonnier, F. , Roger, S. , Guerin, P. , Molinari, F. , M'Rad, R. , Cahard, D. , … Briault, S. (2006). Association of a functional deficit of the BKCa channel, a synaptic regulator of neuronal excitability, with autism and mental retardation. The American Journal of Psychiatry, 163(9), 1622–1629. 10.1176/ajp.2006.163.9.1622 16946189

[brb3978-bib-0062] Lee, E. J. , Choi, S. Y. , & Kim, E. (2015). NMDA receptor dysfunction in autism spectrum disorders. Current Opinion in Pharmacology, 20, 8–13. 10.1016/j.coph.2014.10.007 25636159

[brb3978-bib-0063] Lemonnier, E. , Degrez, C. , Phelep, M. , Tyzio, R. , Josse, F. , Grandgeorge, M. , … Ben‐Ari, Y. (2012). A randomised controlled trial of bumetanide in the treatment of autism in children. Translational Psychiatry, 2, e202 10.1038/tp.2012.124 23233021 PMC3565189

[brb3978-bib-0064] Levy, D. , Ronemus, M. , Yamrom, B. , Lee, Y.‐H. , Leotta, A. , Kendall, J. , … Wigler, M. (2011). Rare de novo and transmitted copy‐number variation in autistic spectrum disorders. Neuron, 70(5), 886–897. 10.1016/j.neuron.2011.05.015 21658582

[brb3978-bib-0065] Li, J. , Nguyen, L. , Gleason, C. , Lotspeich, L. , Spiker, D. , Risch, N. , & Myers, R. M. (2004). Lack of evidence for an association between WNT2 and RELN polymorphisms and autism. American Journal of Medical Genetics Part B: Neuropsychiatric Genetics, 126B(1), 51–57. 10.1002/(ISSN)1096-8628 15048648

[brb3978-bib-0066] Li, J. , You, Y. , Yue, W. , Jia, M. , Yu, H. , Lu, T. , … Zhang, D. (2015). Genetic evidence for possible involvement of the calcium channel gene CACNA1A in autism pathogenesis in Chinese Han population. PLoS One, 10(11), e0142887 10.1371/journal.pone.0142887 26566276 PMC4643966

[brb3978-bib-0067] Li, J. , Zhao, L. , You, Y. , Lu, T. , Jia, M. , Yu, H. , … Wang, L. (2015). Schizophrenia related variants in CACNA1C also confer risk of autism. PLoS One, 10(7), e0133247 10.1371/journal.pone.0133247 26204268 PMC4512676

[brb3978-bib-0068] Li, X. , Zou, H. , & Brown, W. T. (2012). Genes associated with autism spectrum disorder. Brain Research Bulletin, 88(6), 543–552. 10.1016/j.brainresbull.2012.05.017 22688012

[brb3978-bib-0069] Liu, X. , & Takumi, T. (2014). Genomic and genetic aspects of autism spectrum disorder. Biochemical and Biophysical Research Communications, 452(2), 244–253. 10.1016/j.bbrc.2014.08.108 25173933

[brb3978-bib-0070] Loscher, W. (2002). Basic pharmacology of valproate: A review after 35 years of clinical use for the treatment of epilepsy. CNS Drugs, 16(10), 669–694. 10.2165/00023210-200216100-00003 12269861

[brb3978-bib-0071] Lu, A. T. , Dai, X. , Martinez‐Agosto, J. A. , & Cantor, R. M. (2012). Support for calcium channel gene defects in autism spectrum disorders. Molecular Autism, 3(1), 18 10.1186/2040-2392-3-18 23241247 PMC3558437

[brb3978-bib-0072] Ma, X. M. , & Blenis, J. (2009). Molecular mechanisms of mTOR‐mediated translational control. Nature Reviews Molecular Cell Biology, 10(5), 307–318. 10.1038/nrm2672 19339977

[brb3978-bib-0073] Mabb, A. M. , & Ehlers, M. D. (2010). Ubiquitination in postsynaptic function and plasticity. Annual Review of Cell and Developmental Biology, 26, 179–210. 10.1146/annurev-cellbio-100109-104129 PMC3163670 20604708

[brb3978-bib-0074] Marshall, C. R. , Noor, A. , Vincent, J. B. , Lionel, A. C. , Feuk, L. , Skaug, J. , … Scherer, S. W. (2008). Structural variation of chromosomes in autism spectrum disorder. The American Journal of Human Genetics, 82(2), 477–488. 10.1016/j.ajhg.2007.12.009 18252227 PMC2426913

[brb3978-bib-0075] Martin‐Ruiz, C. M. , Lee, M. , Perry, R. H. , Baumann, M. , Court, J. A. , & Perry, E. K. (2004). Molecular analysis of nicotinic receptor expression in autism. Brain Research Molecular Brain Research, 123(1–2), 81–90. 10.1016/j.molbrainres.2004.01.003 15046869

[brb3978-bib-0076] Matsunami, N. , Hadley, D. , Hensel, C. H. , Christensen, G. B. , Kim, C. , Frackelton, E. , … Hakonarson, H. (2013). Identification of rare recurrent copy number variants in high‐risk autism families and their prevalence in a large ASD population. PLoS One, 8(1), e52239 10.1371/journal.pone.0052239 23341896 PMC3544904

[brb3978-bib-0077] McBride, K. L. , Varga, E. A. , Pastore, M. T. , Prior, T. W. , Manickam, K. , Atkin, J. F. , & Herman, G. E. (2010). Confirmation study of PTEN mutations among individuals with autism or developmental delays/mental retardation and macrocephaly. Autism Research, 3(3), 137–141. 10.1002/aur.132 20533527

[brb3978-bib-0078] Merner, N. D. , Mercado, A. , Khanna, A. R. , Hodgkinson, A. , Bruat, V. , Awadalla, P. , … Kahle, K. T. (2016). Gain‐of‐function missense variant in SLC12A2, encoding the bumetanide‐sensitive NKCC1 cotransporter, identified in human schizophrenia. Journal of Psychiatric Research, 77, 22–26. 10.1016/j.jpsychires.2016.02.016 26955005

[brb3978-bib-0079] Mony, T. J. , Lee, J. W. , Dreyfus, C. , DiCicco‐Bloom, E. , & Lee, H. J. (2016). Valproic acid exposure during early postnatal gliogenesis leads to autistic‐like behaviors in rats. Clinical Psychopharmacology and Neuroscience, 14(4), 338–344. 10.9758/cpn.2016.14.4.338 27776385 PMC5083944

[brb3978-bib-0080] Moosmang, S. , Haider, N. , Klugbauer, N. , Adelsberger, H. , Langwieser, N. , Muller, J. , … Kleppisch, T. (2005). Role of hippocampal Cav1.2 Ca^2+^ channels in NMDA receptor‐independent synaptic plasticity and spatial memory. The Journal of Neuroscience, 25(43), 9883–9892. 10.1523/JNEUROSCI.1531-05.2005 16251435 PMC6725564

[brb3978-bib-0081] Morrow, E. M. , Yoo, S.‐Y. , Flavell, S. W. , Kim, T.‐K. , Lin, Y. , Hill, R. S. , … Walsh, C. A. (2008). Identifying autism loci and genes by tracing recent shared ancestry. Science, 321(5886), 218–223. 10.1126/science.1157657 18621663 PMC2586171

[brb3978-bib-0082] Napoli, I. , Mercaldo, V. , Boyl, P. P. , Eleuteri, B. , Zalfa, F. , De Rubeis, S. , … Bagni, C. (2008). The fragile X syndrome protein represses activity‐dependent translation through CYFIP1, a new 4E‐BP. Cell, 134(6), 1042–1054. 10.1016/j.cell.2008.07.031 18805096

[brb3978-bib-0083] Neale, B. M. , Kou, Y. , Liu, L. , Ma'ayan, A. , Samocha, K. E. , Sabo, A. , … Daly, M. J. (2012). Patterns and rates of exonic de novo mutations in autism spectrum disorders. Nature, 485(7397), 242–245. 10.1038/nature11011 22495311 PMC3613847

[brb3978-bib-0084] O'Roak, B. J. , Deriziotis, P. , Lee, C. , Vives, L. , Schwartz, J. J. , Girirajan, S. , … Eichler, E. E. (2011). Exome sequencing in sporadic autism spectrum disorders identifies severe de novo mutations. Nature Genetics, 43(6), 585–589. 10.1038/ng.835 21572417 PMC3115696

[brb3978-bib-0085] O'Roak, B. J. , Vives, L. , Fu, W. , Egertson, J. D. , Stanaway, I. B. , Phelps, I. G. , … Shendure, J. (2012). Multiplex targeted sequencing identifies recurrently mutated genes in autism spectrum disorders. Science, 338(6114), 1619–1622. 10.1126/science.1227764 23160955 PMC3528801

[brb3978-bib-0086] O'Roak, B. J. , Vives, L. , Girirajan, S. , Karakoc, E. , Krumm, N. , Coe, B. P. , … Eichler, E. E. (2012). Sporadic autism exomes reveal a highly interconnected protein network of de novo mutations. Nature, 485(7397), 246–250. 10.1038/nature10989 22495309 PMC3350576

[brb3978-bib-0087] Ozonoff, S. , Young, G. S. , Carter, A. , Messinger, D. , Yirmiya, N. , Zwaigenbaum, L. , … Stone, W. L. (2011). Recurrence risk for autism spectrum disorders: A Baby Siblings Research Consortium study. Pediatrics, 128(3), e488–e495. 21844053 10.1542/peds.2010-2825 PMC3164092

[brb3978-bib-0088] Parellada, M. , Penzol, M. J. , Pina, L. , Moreno, C. , Gonzalez‐Vioque, E. , Zalsman, G. , & Arango, C. (2014). The neurobiology of autism spectrum disorders. European Psychiatry, 29(1), 11–19. 10.1016/j.eurpsy.2013.02.005 24275633

[brb3978-bib-0089] Park, H. R. , Lee, J. M. , Moon, H. E. , Lee, D. S. , Kim, B. N. , Kim, J. , … Paek, S. H. (2016). A short review on the current understanding of autism spectrum disorders. Experimental Neurobiology, 25(1), 1–13. 10.5607/en.2016.25.1.1 26924928 PMC4766109

[brb3978-bib-0090] Persico, A. M. , & Napolioni, V. (2013). Autism genetics. Behavioural Brain Research, 251, 95–112. 10.1016/j.bbr.2013.06.012 23769996

[brb3978-bib-0091] Pinggera, A. , Lieb, A. , Benedetti, B. , Lampert, M. , Monteleone, S. , Liedl, K. R. , … Striessnig, J. (2015). CACNA1D de novo mutations in autism spectrum disorders activate Cav1.3L‐type calcium channels. Biological Psychiatry, 77(9), 816–822. 10.1016/j.biopsych.2014.11.020 25620733 PMC4401440

[brb3978-bib-0092] Pinto, D. , Darvishi, K. , Shi, X. , Rajan, D. , Rigler, D. , Fitzgerald, T. , … Feuk, L. (2011). Comprehensive assessment of array‐based platforms and calling algorithms for detection of copy number variants. Nature Biotechnology, 29(6), 512–520. 10.1038/nbt.1852 PMC3270583 21552272

[brb3978-bib-0093] Pinto, D. , Pagnamenta, A. T. , Klei, L. , Anney, R. , Merico, D. , Regan, R. , … Betancur, C. (2010). Functional impact of global rare copy number variation in autism spectrum disorders. Nature, 466(7304), 368–372. 10.1038/nature09146 20531469 PMC3021798

[brb3978-bib-0094] Posey, D. J. , Kem, D. L. , Swiezy, N. B. , Sweeten, T. L. , Wiegand, R. E. , & McDougle, C. J. (2004). A pilot study of D‐cycloserine in subjects with autistic disorder. The American Journal of Psychiatry, 161(11), 2115–2117. 10.1176/appi.ajp.161.11.2115 15514414

[brb3978-bib-0095] Robert, C. , Pasquier, L. , Cohen, D. , Fradin, M. , Canitano, R. , Damaj, L. , … Tordjman, S. (2017). Role of genetics in the etiology of autistic spectrum disorder: Towards a hierarchical diagnostic strategy. International Journal of Molecular Sciences, 18(3), E618 10.3390/ijms18030618 28287497 PMC5372633

[brb3978-bib-0096] Roberts, J. E. , Tonnsen, B. L. , McCary, L. M. , Caravella, K. E. , & Shinkareva, S. V. (2016). Brief report: Autism symptoms in infants with fragile X syndrome. Journal of Autism and Developmental Disorders, 46, 3830–3837. 10.1007/s10803-016-2903-5 27628938 PMC5112110

[brb3978-bib-0097] Ronald, A. , & Hoekstra, R. (2014). Progress in understanding the causes of autism spectrum disorders and autistic traits: Twin studies from 1977 to the present day In Rhee S. H., & Ronald A. (Eds.), Behavior genetics of psychopathology (pp. 33–65). New York, NY: Springer 10.1007/978-1-4614-9509-3

[brb3978-bib-0098] Ronemus, M. , Iossifov, I. , Levy, D. , & Wigler, M. (2014). The role of de novo mutations in the genetics of autism spectrum disorders. Nature Reviews Genetics, 15(2), 133–141. 10.1038/nrg3585 24430941

[brb3978-bib-0099] Rose, J. , Jin, S. X. , & Craig, A. M. (2009). Heterosynaptic molecular dynamics: Locally induced propagating synaptic accumulation of CaM kinase II. Neuron, 61(3), 351–358. 10.1016/j.neuron.2008.12.030 19217373 PMC2677100

[brb3978-bib-0100] Rosenberg, R. E. , Law, J. K. , Yenokyan, G. , McGready, J. , Kaufmann, W. E. , & Law, P. A. (2009). Characteristics and concordance of autism spectrum disorders among 277 twin pairs. Archives of Pediatrics & Adolescent Medicine, 163(10), 907–914. 10.1001/archpediatrics.2009.98 19805709

[brb3978-bib-0101] Sanders, S. J. , Murtha, M. T. , Gupta, A. R. , Murdoch, J. D. , Raubeson, M. J. , Willsey, A. J. , … State, M. W. (2012). De novo mutations revealed by whole‐exome sequencing are strongly associated with autism. Nature, 485(7397), 237–241. 10.1038/nature10945 22495306 PMC3667984

[brb3978-bib-0102] Sanders Stephan, J. , Ercan‐Sencicek, A. G. , Hus, V. , Luo, R. , Murtha Michael, T. , Moreno‐De‐Luca, D. , … State, M. W. (2011). Multiple recurrent de novo CNVs, including duplications of the 7q11.23 Williams syndrome region, are strongly associated with autism. Neuron, 70(5), 863–885. 10.1016/j.neuron.2011.05.002 21658581 PMC3939065

[brb3978-bib-0103] Santini, A. C. , Pierantoni, G. M. , Gerlini, R. , Iorio, R. , Olabinjo, Y. , Giovane, A. , … Sogos, C. (2014). Glix 13, a new drug acting on glutamatergic pathways in children and animal models of autism spectrum disorders. BioMed Research International, 2014, 234295. 24605324 10.1155/2014/234295 PMC3925524

[brb3978-bib-0104] Sanua, V. D. (1983). Infantile autism and childhood schizophrenia: Review of the issues from the sociocultural point of view. Social Science & Medicine, 17(21), 1633–1651. 10.1016/0277-9536(83)90309-X 6359455

[brb3978-bib-0105] Schratt, G. M. , Nigh, E. A. , Chen, W. G. , Hu, L. , & Greenberg, M. E. (2004). BDNF regulates the translation of a select group of mRNAs by a mammalian target of rapamycin‐phosphatidylinositol 3‐kinase‐dependent pathway during neuronal development. The Journal of Neuroscience, 24(33), 7366–7377. 10.1523/JNEUROSCI.1739-04.2004 15317862 PMC6729778

[brb3978-bib-0106] Sebat, J. , Lakshmi, B. , Malhotra, D. , Troge, J. , Lese‐Martin, C. , Walsh, T. , … Wigler, M. (2007). Strong association of de novo copy number mutations with autism. Science, 316(5823), 445–449. 10.1126/science.1138659 17363630 PMC2993504

[brb3978-bib-0107] Simms, B. A. , & Zamponi, G. W. (2014). Neuronal voltage‐gated calcium channels: Structure, function, and dysfunction. Neuron, 82(1), 24–45. 10.1016/j.neuron.2014.03.016 24698266

[brb3978-bib-0108] Skafidas, E. , Testa, R. , Zantomio, D. , Chana, G. , Everall, I. P. , & Pantelis, C. (2014). Predicting the diagnosis of autism spectrum disorder using gene pathway analysis. Molecular Psychiatry, 19(4), 504–510. 10.1038/mp.2012.126 22965006 PMC3966080

[brb3978-bib-0109] Smith, M. , Flodman, P. L. , Gargus, J. J. , Simon, M. T. , Verrell, K. , Haas, R. , … Wallace, D. C. (2012). Mitochondrial and ion channel gene alterations in autism. Biochimica et Biophysica Acta, 1817(10), 1796–1802. 10.1016/j.bbabio.2012.04.004 22538295 PMC3423964

[brb3978-bib-0110] Splawski, I. , Timothy, K. W. , Sharpe, L. M. , Decher, N. , Kumar, P. , Bloise, R. , … Keating, M. T. (2004). Ca(V)1.2 calcium channel dysfunction causes a multisystem disorder including arrhythmia and autism. Cell, 119(1), 19–31. 10.1016/j.cell.2004.09.011 15454078

[brb3978-bib-0111] Splawski, I. , Yoo, D. S. , Stotz, S. C. , Cherry, A. , Clapham, D. E. , & Keating, M. T. (2006). CACNA1H mutations in autism spectrum disorders. The Journal of Biological Chemistry, 281(31), 22085–22091. 10.1074/jbc.M603316200 16754686

[brb3978-bib-0112] Srivastava, R. K. , Agarwal, M. , & Pundhir, A. (2011). Role of donepezil in autism: Its conduciveness in psychopharmacotherapy. Case Reports in Psychiatry, 2011, 563204. 22937405 10.1155/2011/563204 PMC3420777

[brb3978-bib-0113] Stary, A. , Kudrnac, M. , Beyl, S. , Hohaus, A. , Timin, E. N. , Wolschann, P. , … Hering, S. (2008). Molecular dynamics and mutational analysis of a channelopathy mutation in the IIS6 helix of Ca V 1.2. Channels (Austin), 2(3), 216–223. 10.4161/chan.2.3.6160 18836301 PMC3196984

[brb3978-bib-0114] Szatmari, P. , Jones, M. B. , Zwaigenbaum, L. , & MacLean, J. E. (1998). Genetics of autism: Overview and new directions. Journal of Autism and Developmental Disorders, 28(5), 351–368. 10.1023/A:1026096203946 9813773

[brb3978-bib-0115] Tavassoli, T. , Kolevzon, A. , Wang, A. T. , Curchack‐Lichtin, J. , Halpern, D. , Schwartz, L. , … Buxbaum, J. D. (2014). De novo SCN2A splice site mutation in a boy with Autism spectrum disorder. BMC Medical Genetics, 15, 35 10.1186/1471-2350-15-35 24650168 PMC3994485

[brb3978-bib-0116] Toma, C. , Hervas, A. , Torrico, B. , Balmana, N. , Salgado, M. , Maristany, M. , … Cormand, B. (2013). Analysis of two language‐related genes in autism: A case‐control association study of FOXP2 and CNTNAP2. Psychiatric Genetics, 23(2), 82–85. 10.1097/YPG.0b013e32835d6fc6 23277129

[brb3978-bib-0117] Tremont‐Lukats, I. W. , Megeff, C. , & Backonja, M. M. (2000). Anticonvulsants for neuropathic pain syndromes: Mechanisms of action and place in therapy. Drugs, 60(5), 1029–1052. 10.2165/00003495-200060050-00005 11129121

[brb3978-bib-0118] Uddin, M. , Tammimies, K. , Pellecchia, G. , Alipanahi, B. , Hu, P. , Wang, Z. , … Scherer, S. W. (2014). Brain‐expressed exons under purifying selection are enriched for de novo mutations in autism spectrum disorder. Nature Genetics, 46(7), 742–747. 10.1038/ng.2980 24859339

[brb3978-bib-0119] Urbano, M. , Okwara, L. , Manser, P. , Hartmann, K. , Herndon, A. , & Deutsch, S. I. (2014). A trial of D‐cycloserine to treat stereotypies in older adolescents and young adults with autism spectrum disorder. Clinical Neuropharmacology, 37(3), 69–72. 10.1097/WNF.0000000000000033 24824660 PMC4354861

[brb3978-bib-0120] van Woerden, G. M. , Harris, K. D. , Hojjati, M. R. , Gustin, R. M. , Qiu, S. , de Avila, Freire. R. , … Weeber, E. J. (2007). Rescue of neurological deficits in a mouse model for Angelman syndrome by reduction of alphaCaMKII inhibitory phosphorylation. Nature Neuroscience, 10(3), 280–282. 10.1038/nn1845 17259980

[brb3978-bib-0121] Vandam, L. D. (1987). Some aspects of the history of local anesthesia In Strichartz G. R. (Ed.), Local anesthetics (pp. 1–19). Berlin, Heidelberg, Germany: Springer.

[brb3978-bib-0122] Veeramah, K. R. , O'Brien, J. E. , Meisler, M. H. , Cheng, X. , Dib‐Hajj, S. D. , Waxman, S. G. , … Hammer, M. F. (2012). De novo pathogenic SCN8A mutation identified by whole‐genome sequencing of a family quartet affected by infantile epileptic encephalopathy and SUDEP. American Journal of Human Genetics, 90(3), 502–510. 10.1016/j.ajhg.2012.01.006 22365152 PMC3309181

[brb3978-bib-0123] Voineagu, I. , Wang, X. , Johnston, P. , Lowe, J. K. , Tian, Y. , Horvath, S. , … Geschwind, D. H. (2011). Transcriptomic analysis of autistic brain reveals convergent molecular pathology. Nature, 474(7351), 380–384. 10.1038/nature10110 21614001 PMC3607626

[brb3978-bib-0124] Vorstman, J. A. , Staal, W. G. , van Daalen, E. , van Engeland, H. , Hochstenbach, P. F. , & Franke, L. (2006). Identification of novel autism candidate regions through analysis of reported cytogenetic abnormalities associated with autism. Molecular Psychiatry, 11(1), 1, 18–28. 10.1038/sj.mp.4001781 16205736

[brb3978-bib-0125] Waltes, R. , Duketis, E. , Knapp, M. , Anney, R. J. , Huguet, G. , Schlitt, S. , … Chiocchetti, A. G. (2014). Common variants in genes of the postsynaptic FMRP signalling pathway are risk factors for autism spectrum disorders. Human Genetics, 133(6), 781–792. 10.1007/s00439-013-1416-y 24442360

[brb3978-bib-0126] Weiss, L. A. , Arking, D. E. , The Gene Discovery Project of Johns Hopkins the Autism C (2009). A genome‐wide linkage and association scan reveals novel loci for autism. Nature, 461(7265), 802–808. 10.1038/nature08490 19812673 PMC2772655

[brb3978-bib-0127] Weiss, L. A. , Escayg, A. , Kearney, J. A. , Trudeau, M. , MacDonald, B. T. , Mori, M. , … Meisler, M. H. (2003). Sodium channels SCN1A, SCN2A and SCN3A in familial autism. Molecular Psychiatry, 8(2), 186–194. 10.1038/sj.mp.4001241 12610651

[brb3978-bib-0128] Yang, W. , Liu, J. , Zheng, F. , Jia, M. , Zhao, L. , Lu, T. , & Wang, L. (2013). The evidence for association of ATP2B2 polymorphisms with autism in Chinese Han population. PLoS One, 8(4), e61021 10.1371/journal.pone.0061021 23620727 PMC3631200

[brb3978-bib-0129] Yatsenko, S. A. , Hixson, P. , Roney, E. K. , Scott, D. A. , Schaaf, C. P. , Ng, Y. T. , … Lupski, J. R. (2012). Human subtelomeric copy number gains suggest a DNA replication mechanism for formation: Beyond breakage‐fusion‐bridge for telomere stabilization. Human Genetics, 131(12), 1895–1910. 10.1007/s00439-012-1216-9 22890305 PMC3493700

[brb3978-bib-0130] Yi, F. , Danko, T. , Botelho, S. C. , Patzke, C. , Pak, C. , Wernig, M. , & Südhof, T. C. (2016). Autism‐associated SHANK3 haploinsufficiency causes Ih channelopathy in human neurons. Science, 352(6286), aaf2669 10.1126/science.aaf2669 26966193 PMC4901875

[brb3978-bib-0131] Zamponi, G. W. (2016). Targeting voltage‐gated calcium channels in neurological and psychiatric diseases. Nature Reviews Drug Discovery, 15(1), 19–34. 10.1038/nrd.2015.5 26542451

[brb3978-bib-0132] Zhu, M. , Sun, X. , Chen, X. , Xiao, H. , Duan, M. , & Xu, J. (2017). Impact of gabapentin on neuronal high voltage‐activated Ca^2+^ channel properties of injured‐side axotomized and adjacent uninjured dorsal root ganglions in a rat model of spinal nerve ligation. Experimental and Therapeutic Medicine, 13(3), 851–860. 10.3892/etm.2017.4071 28450909 PMC5403705

